# Transcriptome Analyses of Senecavirus A-Infected PK-15 Cells: RIG-I and IRF7 Are the Important Factors in Inducing Type III Interferons

**DOI:** 10.3389/fmicb.2022.846343

**Published:** 2022-03-04

**Authors:** Kenan Peng, Lishuang Deng, Jianfeng Wei, Jun Zhao, Huidan Deng, Qian Tao, Chaoyuan Jiang, Yubing Zeng, Fei Li, Rubo Zhang, Xiangang Sun, Zhiwen Xu, Ling Zhu

**Affiliations:** ^1^College of Veterinary Medicine, Sichuan Agricultural University, Chengdu, China; ^2^Key Laboratory of Animal Diseases and Human Health of Sichuan Province, Chengdu, China

**Keywords:** Senecavirus A, RNA-Seq, innate immunity, type III interferons, host transcriptional responses, host-pathogen interactions

## Abstract

Senecavirus A (SVA) is a new type of virus related to swine vesicular disease, which results in enormous economic losses worldwide. At present, the host transcriptional responses to SVA infection, host-SVA interactions, and the mechanism of SVA in innate immune modulation are not well understood. This study explores the gene expression profiles of PK-15 cells at 0, 6, 12, 18, 24, 36 h SVA post-infection by RNA sequencing. Our analysis identified 61, 510, 1,584, 2,460, and 2,359 differentially expressed genes (DEGs) in the comparison groups S6 vs. Control, S12 vs. Control, S18 vs. Control, S24 vs. Control, S36 vs. Control, respectively. The reproducibility and repeatability of the results were validated by RT-qPCR, and all DEGs exhibited expression patterns consistent with the RNA-seq results. According to GO enrichment analysis and KEGG pathway analysis of DEGs in different periods after SVA infection, we found that SVA infection significantly modified the host cell gene-expression patterns and the host cells responded in highly specific manners, including response to signal reception and transmission, external biotic stimulus, response to the virus and host immune defense response. Notably, we observed the specific induction of type III interferon IFN-λ1 and IFN-λ3, which indicated that type III interferon plays an important antiviral function in PK-15 cells. Furthermore, our results showed that SVA might be recognized by RIG-I/MDA-5 receptors first after infecting PK-15 cells and then activates downstream IRF7-mediated signaling pathways, causing an increase in the expression of type III interferon. This study could provide important insights into the modulation of host metabolism during SVA infection and provide a strong theoretical basis for a better understanding of the pathogenic mechanism and immune escape mechanism of SVA.

## Introduction

Senecavirus A (SVA), previously known as Seneca valley virus, belongs to the genus *Senecavirus* of the family *Picornaviridae* (Zhang et al., 2015; Segalés et al., 2017). It is a non-enveloped, single-stranded, and positive-sense RNA virus. The virus particle is an icosahedral structure with a diameter of approximately 17–25 nm, and its morphological characteristics are very similar to members of the genus *Cardiovirus* (Oliveira et al., 2017). SVA was first discovered in human fetal retinal cell culture medium in 2002, which may be derived from contaminants in fetal bovine serum or porcine trypsin (Reddy et al., 2007). After that, it was developed as a virus oncolytic agent in human cancer therapy but not associated with sporadic cases of vesicular disease in pigs (Koppers-Lalic and Hoeben, 2011; Poirier et al., 2013; Evans et al., 2018). In 2007, the first positive case of SVA related to porcine idiopathic vesicular disease was reported in Canada (Pasma et al., 2008). And then, relevant American researchers came to the same conclusion and described the lesions caused by SVA for the first time (Singh, 2012). Clinical symptoms of SVA infection are complete or ruptured blister-like lesions in the snouts, oral mucosa, and hoof, and interstitial pneumonia is the main histopathological feature of SVA infected piglets (Reddy et al., 2007; Zhu et al., 2017). Usually, the symptoms of SVA infection cannot be effectively distinguished from those of other vesicular diseases, such as vesicular stomatitis virus (VS.V), swine vesicular disease virus (SVDV), foot-and-mouth disease virus (FMDV), and vesicular exanthema of swine virus (VESV) (Zhang et al., 2018). In recent years, vesicular diseases associated with SVA have been reported in several countries (Pasma et al., 2008; Guo et al., 2016; Saporiti et al., 2017; Sun et al., 2017; Wu et al., 2017; Saeng-Chuto et al., 2018; Arzt et al., 2019). It is worth noting that the clinical symptoms and transmission speed caused by SVA have a further increasing trend (Leme et al., 2019). In addition, more and more recombination phenomena have appeared in SVA strains, which indicated that the SVA virus is evolving in a stronger direction (Guo et al., 2020; Peng et al., 2020).

Innate immunity is the first line of defense for host cells against viral infections and an important prerequisite and basis for adaptive immunity (Unterholzner and Bowie, 2008). When pathogens invade a host cell, pathogen-associated molecular patterns (PAMPs) can be recognized by host cell pattern-recognition receptors (PRRs), primarily consisting of nucleotide-binding and oligomerization domain (NOD)-like receptors (NLRs), retinoic acid-inducible gene (RIG)-I-like receptors (RLRs), toll-like receptors (TLRs), and cytoplasmic DNA sensors. Subsequently, PRRs stimulate signaling that triggers an innate immune response, inducing chemokines, pro-inflammatory cytokines, and interferons. As we all know, type I and type III interferons play an important role in host antiviral immunity, but there are relatively few studies on type III interferons. Wang et al. (2020) found that type I interferons play a critical role in host immune responses against SVA infection at an early stage.

At present, our understanding of SVA is still in its infancy. The mechanisms underlying dynamic cellular responses of PK-15 cells during SVA infection have not been fully elucidated yet. To deeply understand the host’s mechanism against SVA infection at the mRNA level, we investigated the global gene expression profiles of PK-15 cells at 0, 6, 12, 18, 24, 36 h post-infection (hpi) *via* RNA sequencing. Subsequently, differentially expressed genes (DEGs) screening, gene function, and signaling pathway enrichment analysis make contributions to understanding the host antiviral responses of SVA were further performed. In addition, we have also conducted a preliminary study on the mechanism of SVA-induced Type III Interferon production in PK-15 cells. Our study provides a foundation for future systematic explorations of the SVA infection mechanism.

## Materials and Methods

### Cells and Virus

Porcine kidney (PK-15) cells with a 13th passage number were preserved in our laboratory and cultured in Dulbecco’s Modified Eagle Medium (DMEM) (Gibco, America), containing 10% fetal bovine serum (FBS) (Gibco, America). Cells were incubated at 37°C with 5% CO_2_. Senecavirus A strain SVV-SC-01 (GenBank accession number: MH716015) was cultured and propagated in PK-15 cells.

### Viral Infection and RNA Extraction

When PK-15 cells reached a confluence of 70–80% of 6-well plate, they were infected with SVA strain SVV-SC-01 at the multiplicity of infection (MOI) of 2. Meanwhile, the mock-infected groups (control group) were cultured in the same SVA-free cell culture medium. After 1 h incubation at 37°C, the cell culture medium was changed to DMEM containing 2% FBS. After removing the supernatant, PK-15 cells were collected at 6 hpi, 12 hpi, 18 hpi, 24 hpi, 36 hpi. The biomass of six samples (each sample was independently replicated three times) was harvested. According to the instructions, total RNAs from SVA-infected and mock-infected groups were extracted using an RNA extraction kit (Invitrogen, CA, United States). The mRNA from the total RNA was converted into Samples with OD (260/280) ratios in the range of 1.8–2.0, and OD (260/230) ratios from 1.8 to 2.2 met the sequencing requirements for identification using NanoPhotometer spectrophotometer. RNA samples with RNA integrity numbers (RINs) greater than 7 were selected for subsequent RNA sequencing, performed using an Agilent 2100 bioanalyzer.

### cDNA Library Construction and Sequencing

A total amount of 1 μg RNA per sample was used as input material for the RNA sample preparations. Following the manufacturer’s recommendations, sequencing libraries were generated using NEBNext^®^ Ultra™ RNA Library Prep Kit for Illumina^®^ (NEB, United States). Subsequently, the prepared cDNA library was loaded onto Illumina HiSeq™ 4000 platform (Illumina, America) for sequencing. The sequenced raw data were deposited in the NCBI SRA data archive. The BioProject accession number is PRJNA797671, including SRR17631821 to SRR17631838 eighteen objects.

### Quality Control and Read Mapping

High-quality clean reads were screened from raw reads by removing reads containing adapter, ploy-N, and low-quality reads to ensure the accuracy of follow-up analysis results. The error rate (%), Q20 and Q30 values, and GC-content (%) of the resulting high-quality clean reads were evaluated.

Hisat2^1^ was used for sequence alignment, which reflected the sample data’s specific location in the reference genome (ENSEMBL: Link of Reference genome file: http://ftp.ensembl.org/pub/release-99/fasta/sus_scrofa/dna/ and link of reference gene annotation file: http://ftp.ensembl.org/pub/release-99/gtf/sus_scrofa/).

### Differential Expression Analysis

The FPKM (expected number of Fragments Per Kilobase of transcript sequence per Million base pairs sequenced) of each gene was calculated and used to represent the gene expression value. We used DESeq2 software to analyze DEGs in all SVA-infected groups compared with the mock-infected groups and the Benjamini-Hochberg False Discovery Rate (FDR) multiple times testing correction methods. The threshold for differentially expressed gene screening was established to be a value of | log_2_ (fold change in a comparison group)| > 1.0 and FDR < 0.05.

Function classification of DEGs was analyzed using the clusterProfiler 3.4.4 software to identify enriched Gene Ontology (GO) terms (The Gene Ontology Consortium, 2018) and the Kyoto Encyclopedia of Genes and Genomes (KEGG) pathways (Kanehisa et al., 2019). GO terms or KEGG pathways with *P*-values < 0.05 were significantly enriched.

### Preliminary Study on the Mechanism of Senecavirus A Induced Type III Interferon Production in PK-15 Cells

BAY11-7082 (NF-κB inhibitor) and BX795 (IRF3/7 inhibitor) were used to explore the function of NF-κB and IRF3/7 signaling pathways in the expression of type III IFNs induced by SVA. The PK-15 cells were divided into four groups: Negative control group, SVA group, BAY11-7082-treated group, and BX795-treated group. When PK-15 cells reached a confluence of 70–80% of a 6-well plate, the BAY11-7082-treated group and BX795-treated group were pretreated with 5 μM of BAY11-7082 or 0.5 μM of BX795 for 1 h, respectively. And then, PK-15 cells were washed with PBS and infected with SVA at an MOI of 2 (except the negative control group). After 1 h incubation, the BAY11-7082-treated and BX795-treated groups were incubated with DMEM containing 2% FBS and 5 μM BAY11-7082 or 0.5 μM BX795, respectively. Negative control and SVA group were only incubated with DMEM supplemented with 2% FBS. The cells were collected at 24 h for subsequent RT-qPCR analysis and western blotting analysis.

### RT-qPCR Analysis

According to the manufacturer’s instructions, one microgram of RNA from each sample was used to synthesize cDNA using the PrimeScript*™* RT reagent kit with gDNA Eraser (Takara, Japan). The cDNA was diluted 10-fold with nuclease-free water, and then RT-qPCR assays were performed on a LightCycler 96 System (Roche Diagnostics, Switzerland). Each reaction including 10 μL of SYBR Green qPCR Master Mix (Takara, Japan), 2 μL of diluted cDNA, 0.8 μL of each primer (0.4 μM), and 6.4 μL nuclease-free water.

The RT-qPCR program was as follows: 95°C for 30 s; followed by 35 PCR cycles at 95°C for 5 s, 60°C for 30 s, and 72°C for 30 s. Subsequently, a melting curve was built. The β-actin was employed as an internal control gene. The gene expression levels were measured using the 2^–ΔΔCT^ method: ΔΔCT = (CQ_target_–CQ_β –actin_)_SVA_—(CQ_target_–CQ_β –actin_)_MOCK_. Each sample for RT-qPCR was run in triplicate. The data were expressed as means (*n* = 3) ± standard deviations (SD). The Student’s *t*-test was used to evaluate the variability between different groups by SPSS 13.0 software. All primers were designed using the NCBI Primers-BLAST online programs,^2^ and their sequences are listed in Supplementary Table 1.

### Western Blotting Analysis

Briefly, PK-15 cells were incubated on ice with RIPA lysis buffer (Beyotime, China) containing 1 mM PMSF (Beyotime, China) for 30 min. Following centrifugation at 12,000 × g for 15 min at 4°C, the total protein concentration was quantified using a bicinchoninic acid assay kit (Thermo Fisher Scientific, United States). Each track was loaded with the same amount of protein (40 μg) for sodium dodecyl sulfate-polyacrylamide gel electrophoresis. Then the proteins were transferred onto a PVDF membrane at 100 V for 90 min. After the transfer, 5% bovine serum albumin (BSA) dissolved in TBST was used for blocking at 37°C for 2 h, after washing with TBST, incubated with primary antibodies: RIG-I (CST, United States), IRF3 polyclonal antibody (Proteintech, China), IRF7 (Aviva Systems Biology, China), anti-Phospho-IRF7 (Bioss, China), GAPDH (Abcam, United States) overnight at 4°C. After washing with TBST three times, membranes were incubated with anti-Rabbit IgG-HRP (Sangon, China) or anti-Mouse IgG-HRP (Sangon, China) for 2 h. The immunoblot signals were analyzed by Image Lab 6.0.1 software after incubation with ECL Plus (Thermo Fisher Scientific, United States). Band density was quantified using Image J software.

## Results

### Transcriptome Sequencing and Quality Control

To explore the alteration in the transcriptome profile of PK-15 cells during SVA infection, we analyzed PK-15 cells samples at different time points after virus infection, including 6 hpi (S6), 12 hpi (S12), 18 hpi (S18), 24 hpi (S24), and 36 hpi (S36), as well as uninfected PK-15 cells (Control) by next-generation sequencing. After filtering out the reads with adapters, the reads with ambiguous nucleotides, and the reads of low quality, a total of 787, 101, 328 clean reads remained, with approximately 6.0 G bases per sample.

The sequencing error rate of all samples was no more than 0.03%. Q20 percentages of clean data for all samples were higher than 97.45%, Q30 percentages of clean data for all samples were higher than 92.88%, and the GC contents of the clean data for all samples ranged between 50.95 and 52.01% (Supplementary Table 2), which meets the experimental requirements. Subsequently, clean reads were aligned to the swine reference genome, which exhibited that all samples had total mapped rates within the range of 95.82–96.39%, and the ratios of the uniquely mapped reads were > 91.86% (Supplementary Table 3). The above results suggested that sequencing quality was satisfactory for further analysis.

As we all know, biological replicates are very important for RNA-seq experiments. Sample correlation analysis is helpful to screen abnormal samples among biological repeated samples. Therefore, the correlation coefficient (R2) was calculated to quantify the correlation between repeated samples to ensure highly reliable repeatability. As shown in Supplementary Figure 1, the *R*^2^-values of samples in the same group were higher than 0.98, demonstrating the treatments repeatable. The above results showed that the quality of the sequence is highly reliable.

### Screening of Differentially Expressed Genes Upon Senecavirus A Infection

**FIGURE 1:**
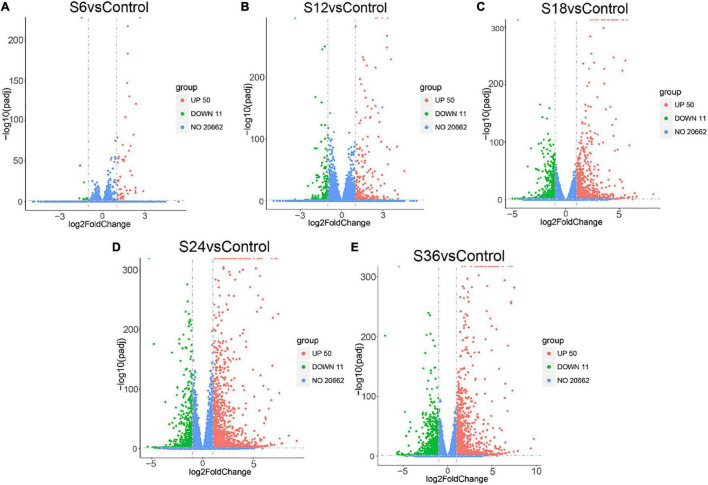
Volcano plots of the DEGs in five comparison groups. Red indicates up-regulated DEGs, green indicates down-regulated DEGs, and blue indicates unchanged DEGs. **(A)** S6 vs. Control. **(B)** S12 vs. Control. **(C)** S18 vs. Control. **(D)** S24 vs. Control. **(E)** S36 vs. Control. Control: PK-15 cells at 0 h SVA post-infection groups; S6_1-3: PK-15 cells at 6 h SVA post-infection groups; S12_1-3: PK-15 cells at 12 h SVA post-infection groups; S18_1-3: PK-15 cells at 18 h SVA post-infection groups; S24_1-3: PK-15 cells at 24 h SVA post-infection groups; S36_1-3: PK-15 cells at 36 h SVA post-infection groups.

Genes that were differentially expressed in each comparison group with | log_2_ (fold change in a comparison group)| > 1.0 and FDR < 0.05 were considered to be DEGs. In general, 61 DEGs were identified in SVA-infected groups at 6 hpi in comparison of the mock-infected group (S6 vs. Control), including 50 up-regulated and 11 down-regulated expression genes (Figure 1A and Supplementary Table 4); For S12 vs. Control, there were 510 DEGs, with 309 genes up-regulated and 201 genes down-regulated (Figure 1B and Supplementary Table 5); For S18 vs. Control, 1,584 DEGs were screened, including 1,023 up-regulated and 561 down-regulated expression genes (Figure 1C and Supplementary Table 6); For S24 vs. Control, 2,460 DEGs were screened, including 1,737 up-regulated and 723 down-regulated expression genes (Figure 1D and Supplementary Table 7); And for S36 vs. Control, 2,359 DEGs were screened, including 1,363 up-regulated and 996 down-regulated expression genes (Figure 1E and Supplementary Table 8). Taken together, compared with the mock-infected group, the total number of DEGs in SVA-infected PK-15 cells gradually increased from 6 hpi and reached the peak at 24 hpi, and then decreased at 36 hpi.

**FIGURE 2:**
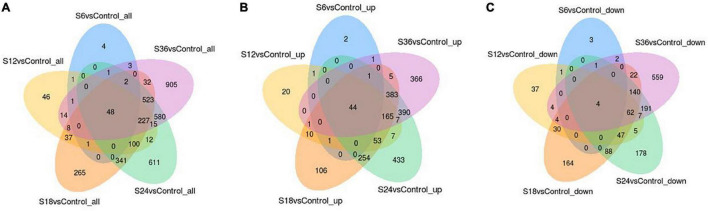
Common genes belonging to DEGs in five comparison groups. **(A)** A total of 48 DEGs were commonly expressed in the five comparison groups. **(B)** 44 Up-regulated DEGs were commonly expressed in the five comparison groups. **(C)** A total of four down-regulated DEGs were commonly expressed in the five comparison groups.

**FIGURE 3:**
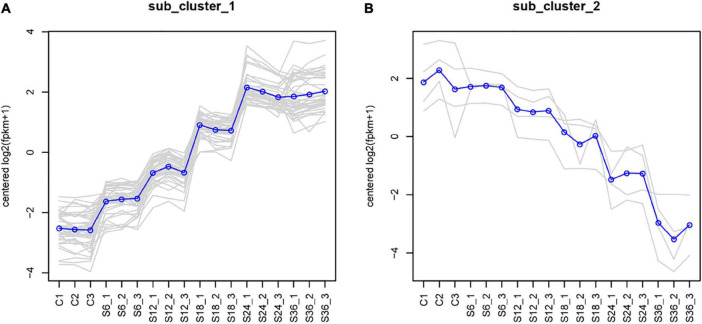
Cluster line chart of five comparison groups. We used the H-cluster method to cluster the DEGs by taking log2 (fpkm + 1) and centralizing correction. The genes in the same cluster had similar expression levels under different treatment conditions. **(A)** DEGs with up-regulated expression trend. **(B)** DEGs with down-regulated expression trend. C1-3: control groups; S6_1-3: PK-15 cells at 6 h SVA post-infection groups; S12_1-3: PK-15 cells at 12 h SVA post-infection groups; S18_1-3: PK-15 cells at 18 h SVA post-infection groups; S24_1-3: PK-15 cells at 24 h SVA post-infection groups; S36_1-3: PK-15 cells at 36 h SVA post-infection groups.

Furthermore, we found that 48 DEGs were commonly expressed in these 5 comparison groups (S6 vs. Control, S12 vs. Control, S18 vs. Control, S24 vs. Control, and S36 vs. Control) (Figure 2A), of which 44 DEGs were up-regulated (Figure 2B and Supplementary Table 9) and 4 DEGs (CYP1A1, IL1RL1, SLC30A1, CYP1B1) were down-regulated (Figure 2C). Significantly, we found that 39 genes showed an upward trend in gene expression with infection time (Figure 3A and Supplementary Table 10), while 4 genes showed a downward trend in gene expression with the passage of infection time (Figure 3B and Supplementary Table 11).

### Gene Ontology Function Enrichment Analysis

The DEGs between each SVA-infected group and the mock-infected group were annotated by the Gene Ontology (GO) function database, which classified into three major categories: biological processes (BP), cellular component (CC), and molecular function (MF). The top 10 GO terms with the most significant enrichment in BP, CC, and MF were selected and displayed in the histogram for each comparison group.

**FIGURE 4:**
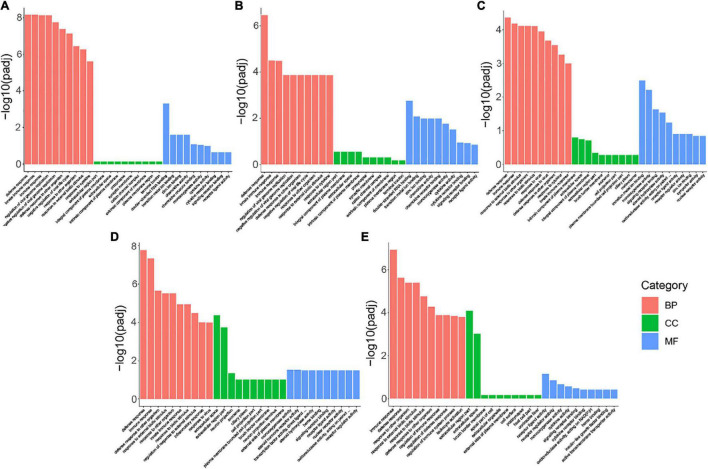
GO function enrichment analysis of DEGs. Significantly enriched GO terms of each category, including biological process (BP), cellular component (CC), and molecular function (MF), were displayed in this figure. **(A)** Enriched GO terms in 6 h vs. Control. **(B)** Enriched GO terms in S12 vs. Control. **(C)** Enriched GO terms in S18 vs. Control. **(D)** Enriched GO terms in S24 vs. Control. **(E)** Enriched GO terms in S36 vs. Control.

DEGs enriched in the BP categories were the most significant in the five comparison groups. Enrichment items were similar, mainly including response to external biotic stimulus, response to another organism, biotic stimulus, response to the virus, defense response to the virus, immune response, and innate immune response (Figure 4). In the MF category, DEGs in S6 vs. Control and S12 vs. Control were primarily associated with GO terms related to double-stranded RNA binding, transition metal ion binding, chemokine activity, and chemokine receptor binding (Figures 4A,B); DEGs in S18 vs. Control were primarily associated with GO terms related to the transition metal ion binding, monooxygenase activity, signaling receptor binding, and steroid hydroxylase activity (Figure 4C); DEGs in S24 vs. Control were primarily associated with GO terms related to monooxygenase activity, steroid hormone receptor activity, transcription factor activity, direct ligand regulated sequence-specific DNA binding, steroid hydroxylase activity, heme binding, signaling receptor binding and receptor-ligand activity (Figure 4D); And the DEGs enriched to the MF categories in S36 vs. Control was associated with receptor-ligand activity (Figure 4E). In the S6 vs. Control, S12 vs. Control, and S18 vs. Control, DEGs enriched in the CC categories were insignificant (Figures 4A–C). In S24 vs. Control and S36 vs. Control, the GO terms with significant enrichment degree in cell component (CC) category were extracellular space and extracellular region part (Figures 4D,E). For more details on GO function enrichment analysis of the five comparison groups, please refer to Supplementary Tables 12–16.

### Kyoto Encyclopedia of Genes and Genomes Pathway Enrichment Analysis

**FIGURE 5:**
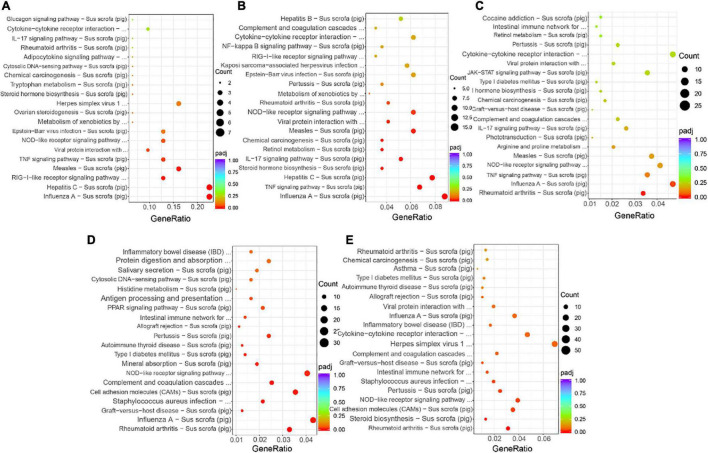
KEGG pathway enrichment analysis. The *x*-axis represents the ratio of the DEGs in this pathway to the total DEGs, and the *y*-axis represents different KEGG pathways. The dot size represents the gene number. **(A)** Top 20 enriched pathways in S6 vs. Control. **(B)** Top 20 enriched pathways in S12 vs. Control. **(C)** Top 20 enriched pathways in S18 vs. Control. **(D)** Top 20 enriched pathways in S24 vs. Control. **(E)** Top 20 enriched pathways in S36 vs. Control.

We performed KEGG annotation and pathway enrichment analysis based on the KEGG database to gain further insight into the functional consequences of gene expression changes associated with SVA infection. We selected the 20 most significant KEGG pathways in the KEGG enrichment results and displayed them in a scatter plot. Figure 5A showed that in S6 vs. Control, DEGs were significantly enriched in the Influenza A, Hepatitis C, RIG-I-like receptor signaling pathway, Measles, TNF signaling pathway, Viral protein interaction with cytokine and cytokine receptor and NOD-like receptor signaling pathway; Pathways in Influenza A, TNF signaling pathway, Hepatitis C, Steroid hormone biosynthesis and IL-17 signaling pathway were mainly enriched in DEGs in S12 vs. Control (Figure 5B); In S18 vs. Control, DEGs were significantly enriched in Rheumatoid arthritis and Influenza A (Figure 5C); In S24 vs. Control, DEGs were significantly enriched in Rheumatoid arthritis, Influenza A and Graft-vs.-host disease and so on (Figure 5D); And in S36 vs. Control, DEGs were mainly significantly enriched in Rheumatoid arthritis, Steroid biosynthesis, Cell adhesion molecules (CAMs) and NOD-like receptor signaling pathway (Figure 5E). For more details on the KEGG pathway enrichment analysis of the five comparison groups, please refer to Supplementary Tables 17–21.

### RT-qPCR Analysis of Differentially Expressed Genes

**FIGURE 6:**
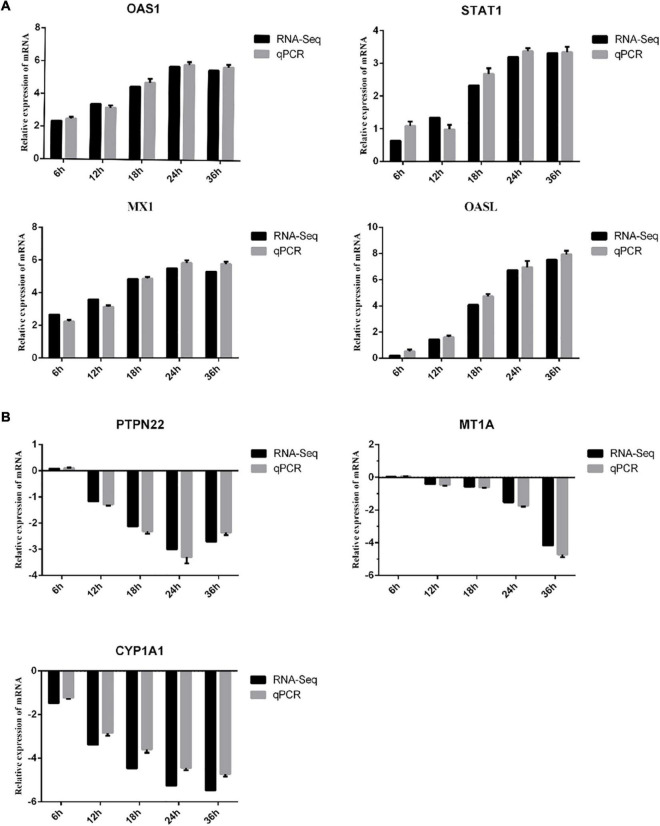
Verification of DEGs by RT-qPCR. The *x*-axis indicates the time post-infection, and the *y*-axis represents the fold changes after SVA infection. **(A)** The up-regulated DEGs. **(B)** The down-regulated DEGs.

To validate the reproducibility and repeatability of DEGs identified from transcriptome sequencing, quantitative reverse transcription PCR (RT-qPCR) was performed. Seven random DEGs, including MX1, OASL, POLQ, OAS1, CYP1A1, PTPN22, and MT1A, were selected for RT-qPCR validation. We found that all 7 DEGs (4 DEGs were up-regulated and 3 DEGs were down-regulated) showed the same expression tendency as RNA-seq analysis (Figure 6).

**FIGURE 7:**
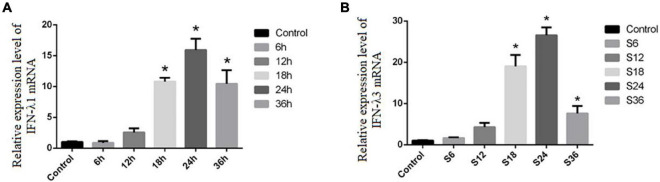
The expression levels of IFN-λ1 and IFN-λ3 in PK-15 after SVA infection. The *x*-axis indicates the time post-infection, and the *y*-axis represents the fold changes after SVA infection (*P < 0.05). **(A)** The mRNA expression of IFN-λ1. **(B)** The mRNA expression of IFN-λ3.

Notably, we find that Type III IFN (including IFN-λ1 and IFN-λ3) showed the largest up-regulation fold change at 18 and 24 hpi, respectively (Supplementary Tables 6, 7). To further verify whether SVA infection induces the production of type III interferon in PK-15 cells, RT-qPCR was used to detect the mRNA level of IFN-λ1 and IFN-λ3 in PK-15 cells following SVA infection for 6, 12, 18, 24, and 36 h (MOI = 2). The results showed that the mRNA expression levels of IFN-λ1 and IFN-λ3 in the SVA-infected group at 18, 24, and 36 h were significantly higher than those in the control group (*P* < 0.05) (Figure 7). These results demonstrated that SVA induced IFN-λ1 and IFN-λ3 production in PK-15 cells.

### Preliminary Study on the Mechanism of Senecavirus A Induced Type III Interferon Production in PK-15 Cells

**FIGURE 8:**
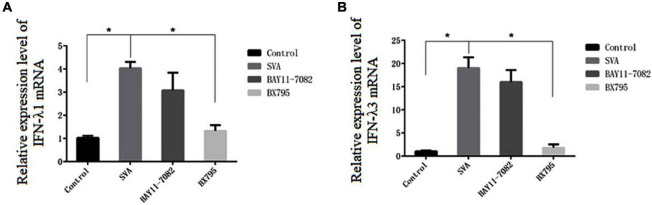
The effect of different inhibitor treatments on type III interferon mRNA expression in SVA-infected PK-15 cells. The *x*-axis represents the different inhibitor treatment groups, and the *y*-axis represents the fold change after SVA infection (*P < 0.05). **(A)** The mRNA expression of IFN-λ1. **(B)** The mRNA expression of IFN-λ3.

Normally, NF-κB and IRF3/IRF7 play an important role in type III IFN production. To identify which transcription factor is associated with type III IFN production after SVA infection in PK-15 cells, the inhibitor BAY11-7082 and BX795 were used. BAY11-7082 inhibits the expression of NF-κB by blocking the phosphorylation of IκBα. BX795 can inhibit the catalysis of TBK1/IKKε kinase from affecting the phosphorylation, nuclear translocation, and transcriptional activity of IRF3/7 and ultimately inhibit the expression of interferon related to this signaling pathway (Clark et al., 2009). As shown in Figure 8, the mRNA expression level of IFN-λ1 and IFN-λ3 in the BX795-treated group decreased compared with the SVA group (*P* < 0.05), whereas no difference was found between the BAY11-7082-treated group and SVA group (*P* > 0.05). These results suggested that SVA may induce type III IFN production *via* the IRF3/7-mediated signaling pathway rather than the NF-κB-mediated signaling pathway.

**FIGURE 9:**
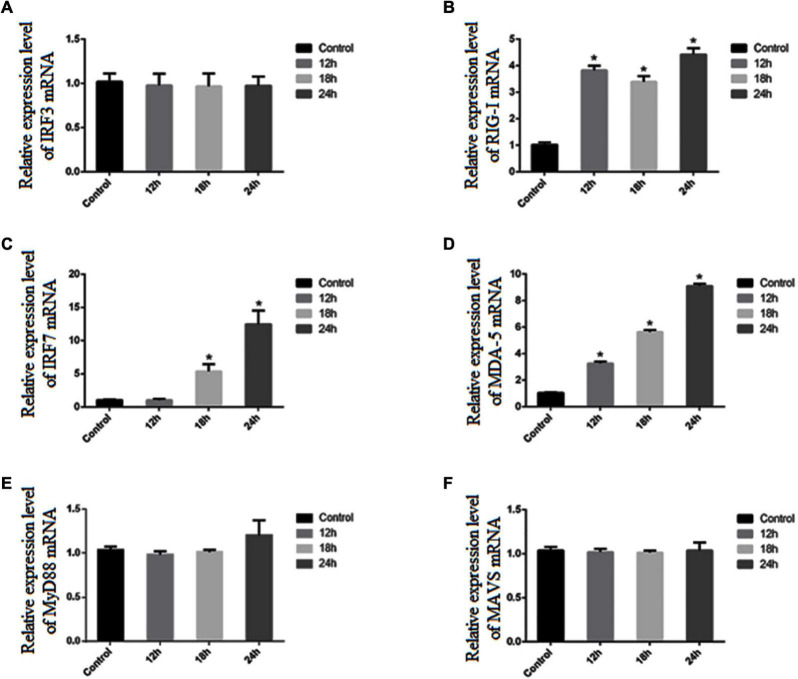
The expression levels of related genes in PK-15 cells after SVA infection. The *x*-axis indicates the time post-infection, and the *y*-axis represents the fold changes after SVA infection (*P < 0.05). **(A)** The mRNA expression of IRF3. **(B)** The mRNA expression of RIG-1. **(C)** The mRNA expression of IRF7. **(D)** The mRNA expression of MDA-5. **(E)** The mRNA expression of MyD88. **(F)** The mRNA expression of MAVS.

**FIGURE 10:**
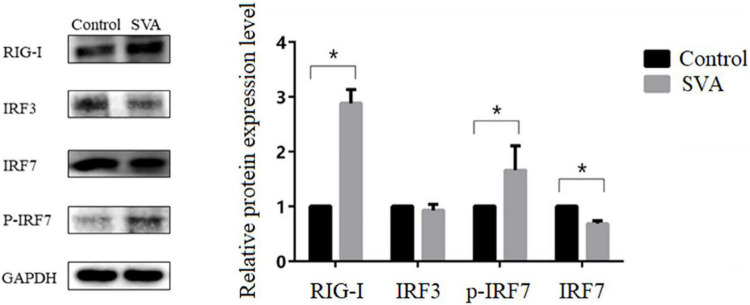
Changes in the protein expression levels of RIG-I, IRF3, IRF7, and p-IRF7 in SVA-infected PK-15 cells. Western blotting was used to assess the expression levels of RIG-I, IRF3, IRF7, and p-IRF7 in the control group and PK-15 cells infected with SVA at an MOI of 2 for 24 h (*P < 0.05).

To further confirm the activation of the IRF3 and IRF7 signaling pathways, RT-qPCR and western blot were used to measure the mRNA and protein expression levels of IRF3 and IRF7 in PK-15 cells after SVA infection. We found that the mRNA expression level of IRF7 was significantly increased at 18 and 24 h after SVA infection (*P* < 0.05), but the mRNA expression level of IRF3 was not significantly increased (*P* > 0.05) (Figures 9A,C). The result of western blot analysis also showed that the expression level of p-IRF7 was significantly higher than that in the control group (*P* < 0.01), while the expression level of IRF7 was down-regulated (*P* < 0.05) (Figure 10). There was no significant change in the expression level of IRF3 (*P* > 0.05) (Figure 10). These results suggested that the expression of type III IFN induced by SVA infection in PK-15 cells mainly depends on IRF7 rather than the IRF3 signaling pathway. Subsequently, we detected the expression level of related receptors (RIG-I, MDA-5) and adaptor proteins (MAVS, MyD88). We found that the mRNA and protein expression levels of RIG-I were significantly increased after SVA infection (*P* < 0.05) (Figures 9B, 10). The mRNA expression level of MDA-5 was significantly increased after SVA infection (*P* < 0.05) (Figure 9D), but the mRNA expression level of MAVS and MyD88 did not change (*P* > 0.05) (Figures 9E,F). Our results suggested that RIG-I and MDA-5 play an important role in the process of SVA-induced type III IFN production in PK-15 cells.

## Discussion

SVA has evolved and spread rapidly as a new swine vesicular disease. It has caused significant economic losses to the global pig industry. Although some progress has been made in studying the mechanism by which SVA exerts immune escape on host cells, many aspects of viruses and diseases have not been fully understood. Further studies are still needed to completely understand the complex molecular mechanism of altering innate immune responses affected by SVA.

RNA-seq and bioinformatics were used to investigate the PK-15 cells treated with SVA for 6, 12, 18, 24, and 36 h (S6, S12, S18, S24, S36) in this study. According to the screening results of DEGs, we found that the number of DEGs was gradually increased with time and reached a peak at 24 h after infection. These results indicated that the expression patterns of genes in the early stage (6, 12 hpi), middle stage (18, 24 hpi), and late stage (36 hpi) after SVA infection were different. According to GO enrichment analysis and KEGG pathway analysis of DEGs in different periods after SVA infection, it can be found that the main biological functions of these DEGs are related to response to signal reception and transmission, external biotic stimulus, response to the virus and host immune defense response. In the early stage of SVA infection, no obvious cytopathic effect (CPE) was observed in PK-15 cells. It indicated that SVA infection did not significantly impact cell growth and metabolism at this stage. Correspondingly, compared with the control group, we found that DEGs enriched in the CC categories were insignificant after SVA infection at 6, 12, and 18 h (Figures 4A–C). The results also confirmed that SVA had little effect on the gene expression of the host cell-related cell cycle, cell metabolism, and cell proliferation in the early stage of infection.

Nevertheless, compared with the control group, DEGs of S24 and S36 enriched in the BP categories were significant and related to extracellular space and extracellular region parts. We speculated that this might be due to the gradual occurrence of CPE in cells in the middle and late stages of SVA infection. In addition, we found that “Influenza A” appeared in the enrichment results of the KEGG signaling pathway of DEGs in multiple comparison groups. Perhaps it is because most of the genes related to “Influenza A” are involved in the process of the antiviral natural immune response, and SVA can also stimulate host cells to produce similar immune regulatory mechanisms.

Viruses usually attack the host cell immune system in the early or middle stages of infection. Therefore, a comprehensive understanding of the changes in the host cell immune system in response to the invasion of external pathogens will help us to clarify better the molecular mechanism of pathogen-host interaction (He et al., 2017; Qi et al., 2017; Lopez et al., 2018; Jiang et al., 2019; Koonpaew et al., 2019). Combining the GO function and the KEGG signal pathway enrichment-analysis results, we found that during SVA infection, DEGs related to chemokine activity, chemokine receptor binding, RIG-I-like receptor signaling pathway, TNF signaling pathway, viral protein interaction with cytokine and cytokine receptor, and NOD-like receptor signaling pathway are involved in the immunological regulation of SVA infection. As we all know, RIG-I-like receptors and NOD-like receptors play an important role in the process of innate immune recognition of viral infection, which is the key to further initiating the host’s innate immune responses. Previous studies have shown that RIG-I plays an important role in host cell recognition of SVA and activation of type I interferon signaling pathway (Li et al., 2018). But the role of the NOD-like receptor signaling pathway in the process of PK-15 cell response to SVA infection still needs further exploration. As an important part of the innate immune system, cytokines are essential for preventing viral infections. There are different types of cytokines, including chemokines, interferons, interleukins, lymphokines, and tumor necrosis factors. And some cytokines can promote immune response by enhancing or inhibiting the effects of other cytokines (Haddad, 2002). Similar to previous results (2020), we found Chemokines (CXCL2 and CCL4), TNF signaling pathway-related factors (TNFAIP3, TNFAIP6, TNFAIP8), and interleukin 6 (IL-6) were up-regulated after SVA infection.

A published study showed that type I IFNs (including IFN-α and IFN-β) was greatly elevated in LLC-PK1 cells with SVA infection (Wang et al., 2020). Interestingly, unlike their findings, we observed the specific type III interferon IFN-λ1 and IFN-λ3 in this study. Both type I interferon and type III interferon can activate the JAK/STAT signaling pathway to induce the expression of interferon-stimulating genes (ISGs), which can directly inhibit virus infection (Haque and Williams, 1998). We found that the expression levels of various ISGs, including MX1, OASL, and ISG-15, were significantly up-regulated during SVA infection. Therefore, we inferred that the increase of these ISGs may be responsible for antiviral effects and inhibition of SVA replication. Therefore, we speculated that type III interferon might play an important role in host immune responses to SVA infection.

It is well known that the expression of type I and type III interferons usually depends on IRF-3/7, NF-κB, MAVS, or other related transcription factors, which are activated by TLRs or RIG-I-dependent signaling pathways (Agalioti et al., 2000; Janeway and Medzhitov, 2002; Honda et al., 2006). RIG-I and MDA-5, a type of DExD/H box helicase, belong to the RIG-I-like receptor family and are mainly responsible for recognizing RNA viruses (Yoneyama et al., 2004, 2005). Previous studies indicated that RIG-I played an antiviral role against SVA and was essential for activating the type I IFN signaling during SVA infection (Li et al., 2018). In this study, we found the mRNA expression level of RIG-I and MDA-5 was significantly increased after SVA infection, indicating that a large amount of RNA produced during the invasion or replication of SVA can be recognized by RIG-I and MDA-5 receptors and then activates downstream related signal pathways to exert innate antiviral immunity. Moreover, we proved that the IRF3/7 signaling pathway is the key factor in inducing the production of type III interferon in PK-15 cells. We did not observe an increase in expression of IRF3, while the mRNA expression level of IRF7 and the protein expression level of p-IRF7 were significantly increased after SVA infection (*P* < 0.05). These results suggested that the expression of type III IFN induced by SVA infection in PK-15 cells mainly depends on IRF7 rather than IRF3. Honda et al. (2005) reported that the transcription factor IRF-7 is essential for the induction of IFN-α/β genes *via* the virus-activated, MyD88-independent pathway and the TLR-activated, MyD88-dependent pathway. Similarly, a previous study showed that although the expression of IFN-λ2 and IFN-λ3 was also regulated by the NF-κB signaling pathway, IRF7 was the main factor regulating their expression (Osterlund et al., 2007). Interestingly, there was no significant increase in MAVS expression, suggesting that the signal transduction mediated by MAVS was not closely related to the mRNA transcription level.

## Conclusion

Our data demonstrated a comprehensive knowledge of gene expression changes in PK-15 infected with SVA in this study. Comprehensive functional analysis of host mRNA profiles revealed that the biological functions of DEGs are mainly related to external stimulus-response, viral response, host immune defense response, and signal reception and conduction. At the same time, we found SVA infection can cause the up-regulation of type III interferon IFN-λ1, IFN-λ3, and related ISGs, which showed type III interferon plays an important role in the host immune response against SVA infection. Then, we conducted a preliminary study on the mechanism of SVA-induced type III interferon production in PK-15 cells. We found that RIG-I/MDA-5 receptors may first recognize SVA after infecting PK-15 cells and then activate downstream IRF7-mediated signaling pathways, causing an increase in the expression of type III interferon. In general, our study could provide important insights into the modulation of host metabolism during SVA infection and provide a strong theoretical basis for a better understanding of the pathogenic mechanism and immune escape mechanism of SVA.

## Data Availability Statement

The data presented in the study are deposited in the NCBI SRA repository, accession number PRJNA797671. The data are also included in the article/Supplementary Material. Further inquiries can be directed to the corresponding author/s.

## Author Contributions

LZ, ZX, and XS contributed to the conception of the study. KP, LD, JW, QT, CJ, YZ, FL, and RZ performed the experiment. KP, LD, and JW contributed significantly to the analysis and manuscript preparation, performed the data analyses, and wrote the manuscript. LZ, ZX, JZ, and HD helped perform the analysis with constructive discussions. All authors contributed to the article and approved the submitted version.

## Conflict of Interest

The authors declare that the research was conducted in the absence of any commercial or financial relationships that could be construed as a potential conflict of interest.

## Publisher’s Note

All claims expressed in this article are solely those of the authors and do not necessarily represent those of their affiliated organizations, or those of the publisher, the editors and the reviewers. Any product that may be evaluated in this article, or claim that may be made by its manufacturer, is not guaranteed or endorsed by the publisher.

## Footnotes

1
http://ccb.jhu.edu/software/hisat2/index.shtml

2
https://www.ncbi.nlm.nih.gov/tools/primer-blast/
