# Supplementary material for: Transcriptome Analyses of Senecavirus A-Infected PK-15 Cells: RIG-I and IRF7 Are the Important Factors in Inducing Type III Interferons
Source: Front Microbiol. 2022 Mar 4;13:846343. doi: 10.3389/fmicb.2022.846343 (PMC8931416; doi:10.3389/fmicb.2022.846343)
Supplement: Supplementary file 1 [file Data_Sheet_1.zip › Supplementary data/Supplementary tables (1-21) and figures.docx]

**Supplementary Table 1** Primers used to verify the selected genes in the RNA-Seq results in this study.

| Gene | Primer sequence (5’-3’) | | Product size (bp) | | Tm (℃) | |
| --- | --- | --- | --- | --- | --- | --- |
| β-actin | F | ATGGACTCTGGGGATGGGG | | 189 | | 60 |
|  | R | CTTCTCCTTGATGTCCCGCA | |  |  |  |
| MX1 | F | CATCTCCAGCCACATCCCTC | | 150 | | 58 |
|  | R | TTCCTCTTGTCGCTGGTGTC | |  |  |  |
| OASL | F | GCTGTGAGGACCGTGAAGAA | | 140 | | 60 |
|  | R | CAGCACAGAAATGCCACCAG | |  |  |  |
| POLQ | F | GTCAACACTGAGCATTCTGGGA | | 132 | | 60 |
|  | R | ATGCAGGCTAAATCCAATGCT | |  |  |  |
| OAS1 | F | CATCCAGGAAATTCGGAGACAG | | 159 | | 59 |
|  | R | GGCAGGACATCAAACTCCACCTC | |  |  |  |
| CYP1A1 | F | CCTCCTTCGTTCCCTTCACC | | 148 | | 59 |
|  | R | CGGAACACAGAGGGGTCATC | |  |  |  |
| PTPN22 | F | CAAGAATGCCGACACAACCA | | 143 | | 60 |
|  | R | TTCCAGCTGCATTAACGGGT | |  |  |  |
| MT1A | F | TCCTGCAAGAAGAGCTGCTG | | 136 | | 60 |
|  | R | ATTTACATCTGGGGCAGGGC | |  |  |  |
| IFN-λ1 | F | AACTTCAGGCTTGCATCAG | | 103 | | 60 |
|  | R | GACTCTTTCTTTGTGGCTTCTT | |  |  |  |
| IFN-λ3 | F | CCAAGGATGCCTTTGAAGAGT | | 178 | | 60 |
|  | R | CTGCTGTGCAGGGATGAGTT | |  |  |  |
| RIG-I | F | ATCCCAGCAACGAGAA | | 188 | | 58 |
|  | R | GCCACGTCCAGTCAAT | |  |  |  |
| MDA5 | F | CCTACGTCCTGGTTGC | | 217 | | 58 |
|  | R | GATGGGTTGTCCTTGC | |  |  |  |
| IRF3 | F | AGAAGCATTGCGTTTAGC | | 300 | | 60 |
|  | R | TCACGGACTCCCAGGTT | |  |  |  |
| IRF7 | F | CCACACTACACCATCTACC | | 197 | | 60 |
|  | R | TCGTCATAGAGGCTGTTG | |  |  |  |
| MAVS | F | ATAGCCAGCCTTTCTCGG | | 237 | | 58 |
|  | R | TAGCCTCAGTCTTGACCTCTTC | |  |  |  |
| MyD88 | F | CGTCGGATGGTAGTGG | | 166 | | 60 |
|  | R | TGATGAACCGCAGGAT | |  |  |  |

**Supplementary Table 2** RNA-Seq sequencing data quality assessment statistics.

| Sample | Raw_reads | Clean_reads | Clean_bases | Error rate（%） | Q20（%） | Q30（%） | GC content（%） |
| --- | --- | --- | --- | --- | --- | --- | --- |
| C1 | 46700820 | 44885714 | 6.73G | 0.03 | 97.51 | 93.04 | 51.54 |
| C2 | 52192402 | 50029382 | 7.5G | 0.03 | 97.56 | 93.13 | 51.79 |
| C3 | 45786176 | 43653506 | 6.55G | 0.03 | 97.56 | 93.15 | 51.5 |
| S6_1 | 49629264 | 47850474 | 7.18G | 0.03 | 97.45 | 92.88 | 51.78 |
| S6_2 | 47753346 | 45256630 | 6.79G | 0.03 | 97.69 | 93.41 | 51.8 |
| S6_3 | 45889440 | 43928538 | 6.59G | 0.03 | 97.62 | 93.28 | 51.55 |
| S12_1 | 40971254 | 39345158 | 5.9G | 0.02 | 98.02 | 94.21 | 51.34 |
| S12_2 | 47071038 | 44904022 | 6.74G | 0.03 | 97.69 | 93.44 | 51.09 |
| S12_3 | 47976358 | 45647996 | 6.85G | 0.03 | 97.65 | 93.34 | 51.21 |
| S18_1 | 47211452 | 45375914 | 6.81G | 0.03 | 97.61 | 93.27 | 51.21 |
| S18_2 | 46278606 | 43892732 | 6.58G | 0.03 | 97.65 | 93.41 | 51.81 |
| S18_3 | 46736716 | 44751384 | 6.71G | 0.03 | 97.57 | 93.2 | 51.74 |
| S24_1 | 47163386 | 45012990 | 6.75G | 0.03 | 97.57 | 93.19 | 51.98 |
| S24_2 | 43639992 | 41363460 | 6.2G | 0.03 | 97.69 | 93.5 | 51.83 |
| S24_3 | 42112724 | 40162622 | 6.02G | 0.03 | 97.58 | 93.19 | 52.01 |
| S36_1 | 42052100 | 39866358 | 5.98G | 0.03 | 97.7 | 93.5 | 51.94 |
| S36_2 | 44427736 | 41654418 | 6.25G | 0.03 | 97.72 | 93.53 | 51.07 |
| S36_3 | 41851108 | 39520030 | 5.93G | 0.03 | 97.71 | 93.46 | 50.95 |

**Supplementary Table 3** Clean reads alignment results.

| Samples | Total_reads | Total_mapped | Unique_mapped | Multiple_mapped |
| --- | --- | --- | --- | --- |
| C1 | 44885714 | 43067810(95.95%) | 41635085(92.76%) | 1432725(3.19%) |
| C2 | 50029382 | 48041152(96.03%) | 46452202(92.85%) | 1588950(3.18%) |
| C3 | 43653506 | 41902147(95.99%) | 40510300(92.8%) | 1391847(3.19%) |
| S6_1 | 47850474 | 45885954(95.89%) | 43964499(91.88%) | 1921455(4.02%) |
| S6_2 | 45256630 | 43493592(96.1%) | 41952363(92.7%) | 1541229(3.41%) |
| S6_3 | 43928538 | 42198172(96.06%) | 40693173(92.63%) | 1504999(3.43%) |
| S12_1 | 39345158 | 37924378(96.39%) | 36535111(92.86%) | 1389267(3.53%) |
| S12_2 | 44904022 | 43098587(95.98%) | 41247896(91.86%) | 1850691(4.12%) |
| S12_3 | 45647996 | 43823078(96.0%) | 42218615(92.49%) | 1604463(3.51%) |
| S18_1 | 45375914 | 43541744(95.96%) | 41949959(92.45%) | 1591785(3.51%) |
| S18_2 | 43892732 | 42117611(95.96%) | 40494360(92.26%) | 1623251(3.7%) |
| S18_3 | 44751384 | 42881896(95.82%) | 41214081(92.1%) | 1667815(3.73%) |
| S24_1 | 45012990 | 43147702(95.86%) | 41457377(92.1%) | 1690325(3.76%) |
| S24_2 | 41363460 | 39708754(96.0%) | 38139697(92.21%) | 1569057(3.79%) |
| S24_3 | 40162622 | 38556789(96.0%) | 37029954(92.2%) | 1526835(3.8%) |
| S36_1 | 39866358 | 38354609(96.21%) | 37023154(92.87%) | 1331455(3.34%) |
| S36_2 | 41654418 | 40018188(96.07%) | 38626423(92.73%) | 1391765(3.34%) |
| S36_3 | 39520030 | 38009640(96.18%) | 36657042(92.76%) | 1352598(3.42%) |

**Supplementary Table** 4 Differentially expressed genes in S6 vs Control.

| Gene_ID | Gene_name | Gene_ID | Gene_name |
| --- | --- | --- | --- |
| ENSSSCG00000012077 | MX1 | ENSSSCG00000046640 | OAS1 |
| ENSSSCG00000001906 | CYP1A1 | ENSSSCG00000034570 | IFI6 |
| ENSSSCG00000009881 | OAS2 | ENSSSCG00000003805 | PDE4B |
| ENSSSCG00000017754 | - | ENSSSCG00000009720 | DDX60 |
| ENSSSCG00000007507 | PCK1 | ENSSSCG00000016254 | - |
| ENSSSCG00000003763 | IFI44 | ENSSSCG00000051209 | - |
| ENSSSCG00000012076 | MX2 | ENSSSCG00000034802 | - |
| ENSSSCG00000040575 | ISG15 | ENSSSCG00000000774 | USP18 |
| ENSSSCG00000017416 | DHX58 | ENSSSCG00000035297 | ISG12(A) |
| ENSSSCG00000008496 | EIF2AK2 | ENSSSCG00000016502 | PARP12 |
| ENSSSCG00000014670 | TRIM22 | ENSSSCG00000033613 | FOXS1 |
| ENSSSCG00000030548 | HERC5 | ENSSSCG00000036956 | SOCS3 |
| ENSSSCG00000007508 | ZBP1 | ENSSSCG00000014565 | - |
| ENSSSCG00000037572 | EPSTI1 | ENSSSCG00000023178 | BATF2 |
| ENSSSCG00000023379 | UBE2L6 | ENSSSCG00000014672 | - |
| ENSSSCG00000008966 | PARM1 | ENSSSCG00000049704 | - |
| ENSSSCG00000004572 | - | ENSSSCG00000008648 | RSAD2 |
| ENSSSCG00000011874 | PARP14 | ENSSSCG00000039472 | SLC30A1 |
| ENSSSCG00000027660 | IFI44L | ENSSSCG00000033844 | CYP1B1 |
| ENSSSCG00000016512 | ZC3HAV1 | ENSSSCG00000029304 | STEAP3 |
| ENSSSCG00000035284 | BMF | ENSSSCG00000001849 | ANPEP |
| ENSSSCG00000010452 | IFIT1 | ENSSSCG00000012027 | ADAMTS5 |
| ENSSSCG00000008959 | CXCL2 | ENSSSCG00000036865 | SDS |
| ENSSSCG00000035037 | - | ENSSSCG00000027877 | TLR10 |
| ENSSSCG00000036383 | LGALS3BP | ENSSSCG00000002001 | REC8 |
| ENSSSCG00000012853 | IRF7 | ENSSSCG00000005385 | NR4A3 |
| ENSSSCG00000039514 | ID3 | novel.365 | - |
| ENSSSCG00000032795 | IL1RL1 | ENSSSCG00000001746 | PKHD1 |
| ENSSSCG00000015897 | IFIH1 | ENSSSCG00000036135 | COL1A1 |
| ENSSSCG00000038912 | IFITM3 | ENSSSCG00000029838 | FZD2 |
| ENSSSCG00000017886 | FBXO39 |  |  |

**Supplementary Table** 5 Differentially expressed genes in S12 vs Control.

| Gene_ID | Gene_name | Gene_ID | Gene_name |
| --- | --- | --- | --- |
| ENSSSCG00000009881 | OAS2 | ENSSSCG00000001912 | PML |
| ENSSSCG00000017754 | - | ENSSSCG00000017886 | FBXO39 |
| ENSSSCG00000007507 | PCK1 | ENSSSCG00000007079 | FLRT3 |
| ENSSSCG00000003763 | IFI44 | ENSSSCG00000017614 | TRIM25 |
| ENSSSCG00000001906 | CYP1A1 | ENSSSCG00000011239 | TRANK1 |
| ENSSSCG00000003694 | EMILIN2 | ENSSSCG00000010452 | IFIT1 |
| ENSSSCG00000007508 | ZBP1 | ENSSSCG00000008648 | RSAD2 |
| ENSSSCG00000033657 | GREM1 | ENSSSCG00000008799 | LIMCH1 |
| ENSSSCG00000012076 | MX2 | ENSSSCG00000038521 | CHAC1 |
| ENSSSCG00000005965 | MYC | ENSSSCG00000012967 | FOSL1 |
| ENSSSCG00000032367 | CEBPD | ENSSSCG00000016784 | ANKH |
| ENSSSCG00000012077 | MX1 | ENSSSCG00000035121 | RHBG |
| ENSSSCG00000006543 | ADAR | ENSSSCG00000012030 | CLDN8 |
| ENSSSCG00000008496 | EIF2AK2 | ENSSSCG00000013551 | C3 |
| ENSSSCG00000011874 | PARP14 | ENSSSCG00000009293 | - |
| ENSSSCG00000016057 | STAT1 | ENSSSCG00000004412 | MICAL1 |
| ENSSSCG00000017146 | - | ENSSSCG00000037572 | EPSTI1 |
| ENSSSCG00000035600 | DNAJC12 | ENSSSCG00000004572 | - |
| ENSSSCG00000016925 | PLK2 | ENSSSCG00000008966 | PARM1 |
| ENSSSCG00000021712 | HERC6 | ENSSSCG00000027372 | SAMD9 |
| ENSSSCG00000036383 | LGALS3BP | ENSSSCG00000004789 | THBS1 |
| ENSSSCG00000006940 | CCN1 | ENSSSCG00000022070 | ZNF143 |
| ENSSSCG00000027660 | IFI44L | ENSSSCG00000030241 | TSC22D3 |
| ENSSSCG00000023379 | UBE2L6 | ENSSSCG00000012050 | RCAN1 |
| ENSSSCG00000017416 | DHX58 | ENSSSCG00000031954 | SLC39A10 |
| ENSSSCG00000033453 | BST2 | ENSSSCG00000035037 | - |
| ENSSSCG00000040746 | LRP2 | ENSSSCG00000015140 | HSPA8 |
| ENSSSCG00000030548 | HERC5 | novel.255 | - |
| ENSSSCG00000014670 | TRIM22 | ENSSSCG00000003079 | - |
| ENSSSCG00000011951 | NFKBIZ | ENSSSCG00000029331 | PALLD |
| ENSSSCG00000038912 | IFITM3 | ENSSSCG00000015897 | IFIH1 |
| ENSSSCG00000010922 | ELF3 | ENSSSCG00000016512 | ZC3HAV1 |
| ENSSSCG00000034570 | IFI6 | ENSSSCG00000046640 | OAS1 |
| ENSSSCG00000049851 | - | ENSSSCG00000000521 | PHLDA1 |
| ENSSSCG00000039862 | TRIB3 | ENSSSCG00000020906 | GHSR |
| ENSSSCG00000029456 | SLC7A1 | ENSSSCG00000014336 | EGR1 |
| ENSSSCG00000033537 | - | ENSSSCG00000021359 | CDC42EP3 |
| ENSSSCG00000039514 | ID3 | ENSSSCG00000011106 | CREM |
| ENSSSCG00000040575 | ISG15 | ENSSSCG00000039770 | SLC6A9 |
| ENSSSCG00000025698 | SERPINE1 | ENSSSCG00000040359 | GOLGA7B |

**Supplementary Table** 6 Differentially expressed genes in S18 vs Control.

| Gene_ID | Gene_name | Gene_ID | Gene_name |
| --- | --- | --- | --- |
| ENSSSCG00000016057 | STAT1 | ENSSSCG00000016512 | ZC3HAV1 |
| ENSSSCG00000040575 | ISG15 | ENSSSCG00000009293 | - |
| ENSSSCG00000012077 | MX1 | ENSSSCG00000033613 | FOXS1 |
| ENSSSCG00000017146 | - | ENSSSCG00000032996 | SLC7A5 |
| ENSSSCG00000012076 | MX2 | ENSSSCG00000014565 | - |
| ENSSSCG00000009881 | OAS2 | ENSSSCG00000017614 | TRIM25 |
| ENSSSCG00000017754 | - | ENSSSCG00000039862 | TRIB3 |
| ENSSSCG00000010452 | IFIT1 | ENSSSCG00000010922 | ELF3 |
| ENSSSCG00000021712 | HERC6 | ENSSSCG00000029456 | SLC7A1 |
| ENSSSCG00000007507 | PCK1 | ENSSSCG00000035297 | ISG12(A) |
| ENSSSCG00000008496 | EIF2AK2 | ENSSSCG00000007864 | GPRC5B |
| ENSSSCG00000038912 | IFITM3 | ENSSSCG00000030108 | ZNFX1 |
| ENSSSCG00000003763 | IFI44 | ENSSSCG00000035634 | PLSCR1 |
| ENSSSCG00000014670 | TRIM22 | ENSSSCG00000014672 | - |
| ENSSSCG00000001906 | CYP1A1 | ENSSSCG00000035600 | DNAJC12 |
| ENSSSCG00000036383 | LGALS3BP | ENSSSCG00000039514 | ID3 |
| ENSSSCG00000033453 | BST2 | ENSSSCG00000005944 | NDRG1 |
| ENSSSCG00000034570 | IFI6 | ENSSSCG00000001963 | EGLN3 |
| ENSSSCG00000007508 | ZBP1 | ENSSSCG00000011951 | NFKBIZ |
| ENSSSCG00000023379 | UBE2L6 | ENSSSCG00000004412 | MICAL1 |
| ENSSSCG00000009720 | DDX60 | ENSSSCG00000023178 | BATF2 |
| ENSSSCG00000030548 | HERC5 | ENSSSCG00000046640 | OAS1 |
| ENSSSCG00000017416 | DHX58 | ENSSSCG00000016420 | INSIG1 |
| ENSSSCG00000011239 | TRANK1 | ENSSSCG00000020906 | GHSR |
| ENSSSCG00000011874 | PARP14 | novel.255 | - |
| ENSSSCG00000032367 | CEBPD | ENSSSCG00000012853 | IRF7 |
| ENSSSCG00000006543 | ADAR | ENSSSCG00000015897 | IFIH1 |
| ENSSSCG00000017886 | FBXO39 | ENSSSCG00000006940 | CCN1 |
| ENSSSCG00000013551 | C3 | ENSSSCG00000030300 | - |
| ENSSSCG00000001912 | PML | ENSSSCG00000033385 | - |
| ENSSSCG00000003694 | EMILIN2 | ENSSSCG00000010575 | PPRC1 |
| ENSSSCG00000049851 | - | ENSSSCG00000006693 | PDZK1 |
| ENSSSCG00000007858 | - | ENSSSCG00000027709 | PARP9 |
| ENSSSCG00000008648 | RSAD2 | ENSSSCG00000014997 | - |
| ENSSSCG00000024388 | BNIP3 | ENSSSCG00000016502 | PARP12 |
| ENSSSCG00000027660 | IFI44L | ENSSSCG00000039658 | - |
| ENSSSCG00000035121 | RHBG | ENSSSCG00000007079 | FLRT3 |
| ENSSSCG00000037572 | EPSTI1 | ENSSSCG00000008449 | SLC3A1 |
| ENSSSCG00000016925 | PLK2 | ENSSSCG00000017420 | CNP |
| ENSSSCG00000040746 | LRP2 | ENSSSCG00000038521 | CHAC1 |

**Supplementary Table** 7 Differentially expressed genes in S24 vs Control.

| Gene_ID | Gene_name | Gene_ID | Gene_name |
| --- | --- | --- | --- |
| ENSSSCG00000010554 | SCD | ENSSSCG00000011874 | PARP14 |
| ENSSSCG00000003694 | EMILIN2 | ENSSSCG00000027660 | IFI44L |
| ENSSSCG00000028814 | SOD3 | ENSSSCG00000027372 | SAMD9 |
| ENSSSCG00000024015 | FADS1 | ENSSSCG00000013551 | C3 |
| ENSSSCG00000021208 | SELENOP | ENSSSCG00000016512 | ZC3HAV1 |
| novel.735 | - | ENSSSCG00000011239 | TRANK1 |
| ENSSSCG00000016057 | STAT1 | ENSSSCG00000039793 | PATJ |
| ENSSSCG00000012077 | MX1 | ENSSSCG00000009293 | - |
| ENSSSCG00000017146 | - | ENSSSCG00000017886 | FBXO39 |
| ENSSSCG00000012076 | MX2 | ENSSSCG00000037572 | EPSTI1 |
| ENSSSCG00000009881 | OAS2 | ENSSSCG00000014997 | - |
| ENSSSCG00000017754 | - | ENSSSCG00000020906 | GHSR |
| ENSSSCG00000010452 | IFIT1 | ENSSSCG00000007858 | - |
| ENSSSCG00000021712 | HERC6 | ENSSSCG00000025618 | TAP1 |
| ENSSSCG00000007507 | PCK1 | ENSSSCG00000017416 | DHX58 |
| ENSSSCG00000031361 | CELSR1 | ENSSSCG00000015549 | RNASEL |
| ENSSSCG00000049851 | - | ENSSSCG00000023178 | BATF2 |
| ENSSSCG00000040746 | LRP2 | ENSSSCG00000003137 | PLEKHA4 |
| ENSSSCG00000008496 | EIF2AK2 | ENSSSCG00000035634 | PLSCR1 |
| ENSSSCG00000038912 | IFITM3 | ENSSSCG00000045508 | CCDC180 |
| ENSSSCG00000006776 | MOV10 | ENSSSCG00000040575 | ISG15 |
| ENSSSCG00000006543 | ADAR | ENSSSCG00000035392 | IGFBP2 |
| ENSSSCG00000016420 | INSIG1 | ENSSSCG00000015897 | IFIH1 |
| ENSSSCG00000032367 | CEBPD | ENSSSCG00000039658 | - |
| ENSSSCG00000017614 | TRIM25 | ENSSSCG00000039348 | H1-0 |
| ENSSSCG00000003763 | IFI44 | novel.581 | - |
| ENSSSCG00000005944 | NDRG1 | ENSSSCG00000011357 | SHISA5 |
| ENSSSCG00000010454 | IFIT5 | ENSSSCG00000013181 | SERPING1 |
| ENSSSCG00000014670 | TRIM22 | ENSSSCG00000014672 | - |
| ENSSSCG00000001906 | CYP1A1 | ENSSSCG00000007864 | GPRC5B |
| ENSSSCG00000036383 | LGALS3BP | ENSSSCG00000039751 | NLRC5 |
| ENSSSCG00000033453 | BST2 | ENSSSCG00000035297 | ISG12(A) |
| ENSSSCG00000034570 | IFI6 | ENSSSCG00000016677 | GARS1 |
| ENSSSCG00000007508 | ZBP1 | ENSSSCG00000016263 | - |
| ENSSSCG00000023379 | UBE2L6 | ENSSSCG00000006995 | ASAH1 |
| ENSSSCG00000030548 | HERC5 | ENSSSCG00000026044 | FDFT1 |
| ENSSSCG00000008449 | SLC3A1 | ENSSSCG00000046640 | OAS1 |
| ENSSSCG00000035121 | RHBG | ENSSSCG00000006693 | PDZK1 |
| ENSSSCG00000001912 | PML | ENSSSCG00000015340 | ASNS |
| ENSSSCG00000024388 | BNIP3 | ENSSSCG00000033613 | FOXS1 |

**Supplementary Table** 8 Differentially expressed genes in S36 vs Control.

| Gene_ID | Gene_name | Gene_ID | Gene_name |
| --- | --- | --- | --- |
| ENSSSCG00000003694 | EMILIN2 | ENSSSCG00000010554 | SCD |
| ENSSSCG00000016057 | STAT1 | ENSSSCG00000017087 | GM2A |
| ENSSSCG00000040575 | ISG15 | ENSSSCG00000006776 | MOV10 |
| ENSSSCG00000012077 | MX1 | ENSSSCG00000030108 | ZNFX1 |
| ENSSSCG00000017146 | - | ENSSSCG00000009720 | DDX60 |
| ENSSSCG00000012076 | MX2 | ENSSSCG00000008648 | RSAD2 |
| ENSSSCG00000011848 | TFRC | ENSSSCG00000015657 | PIGR |
| ENSSSCG00000009881 | OAS2 | ENSSSCG00000024015 | FADS1 |
| ENSSSCG00000017754 | - | ENSSSCG00000039348 | H1-0 |
| ENSSSCG00000021712 | HERC6 | ENSSSCG00000017614 | TRIM25 |
| ENSSSCG00000049851 | - | ENSSSCG00000017477 | CASC3 |
| ENSSSCG00000008496 | EIF2AK2 | ENSSSCG00000006543 | ADAR |
| ENSSSCG00000038912 | IFITM3 | ENSSSCG00000008857 | MSMO1 |
| ENSSSCG00000003763 | IFI44 | ENSSSCG00000027860 | ERAP2 |
| ENSSSCG00000014670 | TRIM22 | ENSSSCG00000017420 | CNP |
| ENSSSCG00000001906 | CYP1A1 | ENSSSCG00000031781 | PSMB10 |
| ENSSSCG00000036383 | LGALS3BP | ENSSSCG00000016872 | HMGCS1 |
| ENSSSCG00000033453 | BST2 | ENSSSCG00000024973 | - |
| ENSSSCG00000034570 | IFI6 | ENSSSCG00000035634 | PLSCR1 |
| ENSSSCG00000007508 | ZBP1 | ENSSSCG00000005944 | NDRG1 |
| ENSSSCG00000023379 | UBE2L6 | ENSSSCG00000046640 | OAS1 |
| ENSSSCG00000030548 | HERC5 | ENSSSCG00000013072 | FADS2 |
| ENSSSCG00000001912 | PML | ENSSSCG00000018084 | ND3 |
| ENSSSCG00000017416 | DHX58 | ENSSSCG00000010493 | PDLIM1 |
| ENSSSCG00000027660 | IFI44L | ENSSSCG00000030300 | - |
| ENSSSCG00000013551 | C3 | ENSSSCG00000008261 | HK2 |
| ENSSSCG00000012853 | IRF7 | ENSSSCG00000010432 | ASAH2 |
| ENSSSCG00000017886 | FBXO39 | ENSSSCG00000016420 | INSIG1 |
| ENSSSCG00000013181 | SERPING1 | ENSSSCG00000016502 | PARP12 |
| ENSSSCG00000014565 | - | ENSSSCG00000004687 | B2M |
| novel.581 | - | ENSSSCG00000016027 | ITGAV |
| ENSSSCG00000037572 | EPSTI1 | ENSSSCG00000002383 | FOS |
| ENSSSCG00000035121 | RHBG | ENSSSCG00000010452 | IFIT1 |
| ENSSSCG00000035297 | ISG12(A) | ENSSSCG00000000697 | - |
| ENSSSCG00000033613 | FOXS1 | ENSSSCG00000007507 | PCK1 |
| ENSSSCG00000014080 | HMGCR | ENSSSCG00000024388 | BNIP3 |
| ENSSSCG00000030277 | - | ENSSSCG00000010454 | IFIT5 |
| ENSSSCG00000009921 | OASL | ENSSSCG00000014672 | - |
| ENSSSCG00000025618 | TAP1 | ENSSSCG00000026044 | FDFT1 |
| ENSSSCG00000031888 | DDIT4 | ENSSSCG00000009240 | - |

**Supplementary Table** 9 List of common up-regulated DEGs.

| Gene ID | Gene | Gene ID | Gene |
| --- | --- | --- | --- |
| ENSSSCG00000012077 | MX1 | ENSSSCG00000011874 | PARP14 |
| ENSSSCG00000009881 | OAS2 | ENSSSCG00000027660 | IFI44L |
| ENSSSCG00000007507 | PCK1 | ENSSSCG00000016512 | ZC3HAV1 |
| ENSSSCG00000003763 | IFI44 | ENSSSCG00000010452 | IFIT1 |
| ENSSSCG00000012076 | MX2 | ENSSSCG00000008959 | CXCL2 |
| ENSSSCG00000040575 | ISG15 | ENSSSCG00000036383 | LGALS3BP |
| ENSSSCG00000017416 | DHX58 | ENSSSCG00000039514 | ID3 |
| ENSSSCG00000008496 | EIF2AK2 | ENSSSCG00000015897 | IFIH1 |
| ENSSSCG00000014670 | TRIM22 | ENSSSCG00000038912 | IFITM3 |
| ENSSSCG00000030548 | HERC5 | ENSSSCG00000017886 | FBXO39 |
| ENSSSCG00000007508 | ZBP1 | ENSSSCG00000046640 | OAS1 |
| ENSSSCG00000037572 | EPSTI1 | ENSSSCG00000034570 | IFI6 |
| ENSSSCG00000023379 | UBE2L6 | ENSSSCG00000009720 | DDX60 |
| ENSSSCG00000008966 | PARM1 | ENSSSCG00000000774 | USP18 |
| ENSSSCG00000033613 | FOXS1 | ENSSSCG00000035297 | ISG12(A) |
| ENSSSCG00000036956 | SOCS3 | ENSSSCG00000016502 | PARP12 |
| ENSSSCG00000008648 | RSAD2 | ENSSSCG00000002001 | REC8 |
| ENSSSCG00000023178 | BATF2 | ENSSSCG00000004572 | - |
| ENSSSCG00000017754 | - | ENSSSCG00000016254 | - |
| ENSSSCG00000014565 | - | ENSSSCG00000035037 | - |
| ENSSSCG00000014672 | - | ENSSSCG00000051209 | - |
| ENSSSCG00000049704 | - | ENSSSCG00000034802 | - |

**Supplementary Table** 10 DEGs with up-regulated expression trend.

| Gene_ID | Gene_name | Gene_ID | Gene_name |
| --- | --- | --- | --- |
| NSSSCG00000012077 | MX1 | ENSSSCG00000009720 | DDX60 |
| ENSSSCG00000009881 | OAS2 | ENSSSCG00000034802 | - |
| ENSSSCG00000017754 | - | ENSSSCG00000000774 | USP18 |
| ENSSSCG00000003763 | IFI44 | ENSSSCG00000035297 | ISG12(A) |
| ENSSSCG00000012076 | MX2 | ENSSSCG00000033613 | FOXS1 |
| ENSSSCG00000040575 | ISG15 | ENSSSCG00000014565 | - |
| ENSSSCG00000017416 | DHX58 | ENSSSCG00000014672 | - |
| ENSSSCG00000017146 | - | ENSSSCG00000008648 | RSAD2 |
| ENSSSCG00000014670 | TRIM22 | ENSSSCG00000014997 | - |
| ENSSSCG00000030548 | HERC5 | ENSSSCG00000013551 | C3 |
| ENSSSCG00000007508 | ZBP1 | ENSSSCG00000007858 | - |
| ENSSSCG00000021712 | HERC6 | ENSSSCG00000010451 | IFIT2 |
| ENSSSCG00000027660 | IFI44L | novel.581 | - |
| ENSSSCG00000010452 | IFIT1 | ENSSSCG00000032474 | CXCL10 |
| ENSSSCG00000036383 | LGALS3BP | ENSSSCG00000032436 | - |
| ENSSSCG00000033453 | BST2 | ENSSSCG00000001636 | - |
| ENSSSCG00000038912 | IFITM3 | novel.598 | - |
| ENSSSCG00000017886 | FBXO39 | ENSSSCG00000009921 | OASL |
| ENSSSCG00000046640 | OAS1 | novel.221 | - |
| ENSSSCG00000034570 | IFI6 |  |  |

**Supplementary Table** 11 DEGs with down-regulated expression trend.

| Gene_ID | Gene_name |
| --- | --- |
| ENSSSCG00000001906 | CYP1A1 |
| ENSSSCG00000030300 | - |
| ENSSSCG00000023684 | MT1A |
| ENSSSCG00000023305 | - |

**Supplementary Table** 12 GO enrichment results of S6 vs Control.

| Category | Gene Name | Up-regulated number | Down-regulated number |
| --- | --- | --- | --- |
| BP | OAS2/PCK1/IFI44/DHX58/EIF2AK2/HERC5/CXCL2/IRF7/IFIH1/IFI6/PDE4B/-/RSAD2 | 13 | 0 |
| BP | OAS2/PCK1/IFI44/DHX58/EIF2AK2/HERC5/CXCL2/IRF7/IFIH1/IFI6/PDE4B/-/RSAD2 | 13 | 0 |
| BP | OAS2/PCK1/IFI44/DHX58/EIF2AK2/HERC5/CXCL2/IRF7/IFIH1/IFI6/PDE4B/-/RSAD2 | 13 | 0 |
| BP | OAS2/DHX58/EIF2AK2/HERC5/IRF7/IFIH1/IFI6/-/RSAD2 | 9 | 0 |
| BP | OAS2/DHX58/HERC5/IRF7/IFIH1/IFI6/-/RSAD2 | 8 | 0 |
| BP | OAS2/DHX58/HERC5/ZBP1/CXCL2/IRF7/IL1RL1/IFIH1/IFI6/-/SOCS3/-/RSAD2/TLR10 | 12 | 2 |
| BP | OAS2/IFI44/DHX58/ZBP1/CXCL2/IRF7/IL1RL1/IFIH1/IFI6/PDE4B/-/-/RSAD2/TLR10 | 12 | 2 |
| BP | OAS2/DHX58/ZBP1/IRF7/IFIH1/IFI6/-/-/RSAD2/TLR10 | 9 | 1 |
| BP | -/DHX58/EIF2AK2/IRF7/IL1RL1/IFIH1/PDE4B/RSAD2/CYP1B1 | 7 | 2 |
| BP | -/DHX58/EIF2AK2/IRF7/IL1RL1/IFIH1/PDE4B/RSAD2/CYP1B1/TLR10 | 7 | 3 |
| BP | OAS2/DHX58/HERC5/IRF7/IFIH1/IFI6/-/RSAD2 | 8 | 0 |
| BP | -/DHX58/EIF2AK2/IRF7/IL1RL1/IFIH1/PDE4B/RSAD2/CYP1B1/TLR10 | 7 | 3 |
| BP | DHX58/IL1RL1/TLR10 | 1 | 2 |
| BP | CXCL2/- | 2 | 0 |
| BP | IRF7/IL1RL1/RSAD2 | 2 | 1 |
| BP | -/IL1RL1/IFIH1 | 2 | 1 |
| BP | PCK1/CXCL2/ID3/PDE4B | 4 | 0 |
| MF | OAS2/DHX58/EIF2AK2/IFIH1 | 4 | 0 |
| MF | PCK1/DHX58/TRIM22/IFIH1/FBXO39/-/CYP1B1 | 6 | 1 |
| MF | CXCL2/- | 2 | 0 |
| MF | CXCL2/- | 2 | 0 |

**Supplementary Table** 13 GO enrichment results of S12 vs Control.

| Category | Gene name | Up-regulated number | Down-regulated number |
| --- | --- | --- | --- |
| BP | OAS2/ZBP1/ADAR/DHX58/HERC5/ELF3/IFI6/SERPINE1/RSAD2/C3/THBS1/IFIH1/CXCL2/IL1RL1/-/ZC3H12A/TRIM21/-/S100A12/-/TLR4/IL22RA1/SOCS3/DRD1/DDX58/S100A8/OASL/DUOX2/TCIRG1/-  /SOCS1/CCL4/CCL1/ACOD1 | 29 | 5 |
| BP | OAS2/ZBP1/ADAR/DHX58/IFI6/RSAD2/C3/IFIH1/-/TRIM21/-/S100A12/-/TLR4/DDX58/S100A8/-/SOCS1/CCL4/CCL1 | 19 | 1 |
| BP | OAS2/IFI44/ZBP1/ADAR/DHX58/IFI6/RSAD2/C3/THBS1/SLC39A10/IFIH1/CXCL2/IL1RL1/-/ZC3H12A/TRIM21/-/S100A12/-/TLR4/TNFSF15/DDX58/S100A8/OASL/CDH17/TCIRG1/-/SOCS1/CCL4/CCL1/ACOD1 | 28 | 3 |
| BP | OAS2/ADAR/EIF2AK2/RSAD2/HSPA8/-/ZC3H12A/OASL | 8 | 0 |
| BP | OAS2/EIF2AK2/RSAD2/-/ZC3H12A/OASL | 6 | 0 |
| BP | OAS2/DHX58/HERC5/IFI6/SERPINE1/RSAD2/IFIH1/-/ZC3H12A/S100A12/-/TLR4/IL22RA1/DDX58/OASL | 14 | 1 |
| BP | OAS2/EIF2AK2/RSAD2/-/ZC3H12A/TRIM21/OASL | 7 | 0 |
| BP | OAS2/SERPINE1/THBS1/TLR4/SHAS2/HABP2/THBD/CPB2 | 5 | 3 |
| BP | ZBP1/DHX58/SERPINE1/RSAD2/C3/THBS1/SLC39A10/IFIH1/IL1RL1/KITLG/ZC3H12A/TLR4/DDX58/-/SOCS1/ACOD1 | 12 | 4 |
| BP | DHX58/HERC5/ZC3H12A/DDX58/ACOD1 | 5 | 0 |
| BP | TRIB3/C3/DRD1/SLC2A5/- | 3 | 2 |
| MF | OAS2/ADAR/EIF2AK2/DHX58/IFIH1/DDX58/OASL | 7 | 0 |
| MF | PCK1/DHX58/TRIM22/FBXO39/IFIH1/EGR1/-/TRIM21/NR4A1/SUOX/TRIM47/S100A12/ERAP2/CYP1B1/APOBEC3B/NEIL2/DDX58/CYP24A1/S100A8/MEP1B/THRB/-/NANOS1/ALOX15B/CPB2 | 20 | 5 |
| MF | DHX58/TRIM22/FBXO39/IFIH1/EGR1/-/TRIM21/NR4A1/TRIM47/S100A12/ERAP2/APOBEC3B/NEIL2/DDX58/S100A8/MEP1B/THRB/NANOS1/CPB2 | 16 | 3 |
| MF | CXCL2/-/CCL4/CCL1 | 3 | 1 |
| MF | CXCL2/-/CCL4/CCL1 | 3 | 1 |
| MF | MICAL1/FMO5/CYP1B1/CYP24A1/-/TYRP1 | 3 | 3 |
| MF | GREM1/CXCL2/KITLG/-/TNFSF15/CCL4/CCL1 | 4 | 3 |

**Supplementary Table** 14 GO enrichment results of S18 vs Control.

| Category | Gene name | Up-regulated number | Down-regulated number |
| --- | --- | --- | --- |
| BP | OAS2/IFI6/ZBP1/HERC5/DHX58/ADAR/C3/RSAD2/-/ELF3/IRF7/IFIH1/-/TAP1/THBS1/ACP5/MOV10/TRIM21/ZC3H12A/DDIT4/NLRC5/SARM1/EREG/IL1RL1/-/OASL/SLC7A2/CXCL2/S100A12/IL22RA1/STAT2/IRF1/TRIM13/TLR4/PTPN6/-/DRD1/ZFP36/SOCS3/TSPAN32/SOCS1/TCIRG1/P2RX7/RASGRP1/METRNL/ALPK1/S100A8/AGBL4/PPARG/IL1A/STING1/ACOD1/DUOX2/DUOXA2/-/-/-/NR1H4/CXCL11/IL28B/ACKR2/IL1R2/GGT5/CCL1 | 56 | 8 |
| BP | OAS2/IFI44/IFI6/ZBP1/DHX58/ADAR/C3/RSAD2/-/IRF7/IFIH1/-/JAG1/THBS1/TRIM21/ZC3H12A/NLRC5/SARM1/EREG/IL1RL1/IGF1R/-/OASL/SLC39A10/EMP2/CXCL2/B2M/S100A12/STAT2/PARP3/DOCK2/-/SPNS2/IRF1/TRIM13/TLR4/PTPN6/ENPP2/-/SOCS1/SMAD6/TCIRG1/P2RX7/RASGRP1/ALPK1/S100A8/TNFSF15/PPARG/IL1A/STING1/ACOD1/CDH17/-/-/-/-/CD38/NR1H4/CXCL11/IL28B/ACKR2/CD274/PDCD1/CCL1/-/LAX1 | 56 | 10 |
| BP | PCK1/DDIT4/S100A12/CRYZ/CYP1B1/-/CYP2F1/ADCY8/CDO1/CYP2C42 | 9 | 1 |
| BP | ACP5/ZC3H12A/SERPINB1/TLR4/TMEM106A/P2RX7/NR1H4/IL1R2 | 8 | 0 |
| BP | ACP5/ZC3H12A/SLC7A2/KLF4/TLR4/CYP1B1/-/NOS1 | 5 | 3 |
| BP | OAS2/PCK1/IFI44/DHX58/C3/-/ACP5/ZC3H12A/NLRC5/NEXN/-/CXCL2/IL22RA1/-/TUT4/TLR4/ZFP36/SMAD6/P2RX7/IL1A/ACOD1/-/NR1H4/CXCL11/CD274/GGT5/NOS1 | 25 | 2 |
| BP | FLRT3/THBS1/ENAH/NEXN/MTUS1/CXCL2/SEMA4B/EFNA1/DOCK2/PREX1/SEMA6A/ENPP2/-/-/ID2/EFNA4/SLC8B1/GBX1/PLXNB3/S100A8/EPHA3/FGF18/CXCL11/CDK5R1/ACKR2/CCL1/- | 17 | 10 |
| BP | PCK1/SMOX/ACP5/HSPA8/ZC3H12A/MDH1/DDIT4/GPX3/DAO/SLC7A2/MPST/KLF4/ATP6V1B1/QDPR/FAH/TLR4/TMEM106A/AK1/DRD1/HAO2/ADA2/BEND3/P2RX7/CYP1B1/DUOX2/DUOXA2/TYRP1/-/HAL/FBP1/CYP2F1/-/GGT5/NOS1/CYP2C42 | 29 | 6 |
| MF | PCK1/TRIM22/DHX58/FBXO39/-/IFIH1/-/TRIM56/ACP5/SOD3/TRIM21/SRSF6/DYRK2/S100A12/MSMO1/ERAP2/RIOX1/MELTF/SUOX/APOBEC3B/NR4A1/CRYZ/TRIM13/TUT4/RNF138/TRIM47/ENPP2/RCHY1/HNF4G/ADA2/CYP39A1/NEIL2/RASGRP1/CYP7A1/S100A8/AGBL4/MEP1B/CYP1B1/ZNF385A/-/PPARG/IL1A/-/APOBEC1/ZCCHC4/CYP24A1/DMD/ERI2/DTX1/-/-/THRB/NR1H4/F5/CYP2F1/CPB2/ZCCHC24/CDO1/CYP2C42/PPM1N | 42 | 18 |
| MF | MICAL1/FMO5/MSMO1/CYP39A1/CYP7A1/CYP1B1/CYP24A1/TYRP1/-/CYP2F1/NOS1/CYP2C42 | 9 | 3 |
| MF | C3/FLRT3/CHAC1/GREM1/-/TAP1/JAG1/THBS1/CCN5/KITLG/HSPA8/SYTL2/EREG/IGF1R/OSGIN1/EMP2/CXCL2/S100A12/SEMA4B/EFNA1/WIPI1/DOCK2/LYNX1/-/NR4A1/SYT1/SEMA6A/TLR4/MTSS1/ADM/COL4A3/FRK/-/ASXL3/EFNA4/ADA2/SOCS1/SMAD6/NKX3-1/DMTN/METRNL/S100A8/ARNTL/-/TNFSF15/CCN2/PPARG/TSPOAP1/IL1A/CDH17/PTHLH/FGF18/DTX1/-/-/NR1H4/CXCL11/IL28B/CDK5R1/NLGN3/WNT6/CCL1/- | 47 | 16 |
| MF | CYP39A1/CYP7A1/CYP1B1/-/CYP2F1/CYP2C42 | 5 | 1 |

**Supplementary Table** 15 GO enrichment results of S24 vs Control.

| Category | Gene name | Up-regulated number | Down-regulated number |
| --- | --- | --- | --- |
| BP | OAS2/MOV10/ADAR/IFI6/ZBP1/HERC5/C3/TAP1/DHX58/IFIH1/NLRC5/RSAD2/THBS1/IRF7/ELF3/TRIM21/ACP5/IFNAR1/DPP4/-/STAT2/SARM1/IL22RA1/-/-/OASL/IL1RL1/ZC3H12A/DDIT4/ZFP36/S100A12/LGALS8/CXCL2/CADM1/UNC93B1/IRF1/TRAF3IP2/SLC7A2/SOCS1/TRIM13/TCIRG1/ALPK1/IRAK2/P2RX7/TLR4/RASGRP1/BST1/MFHAS1/BCL3/PLA2G6/DUOX2/SOCS3/AGBL4/STING1/TSPAN32/CXCL11/-/S100A8/IL1A/DRD1/-/NR1D1/TYRO3/ACOD1/PTGFR/-/PPARG/UNC13D/SLPI/ACKR2/F12/DDX58/DUOXA2/-/NLRC3/-/C5/NR1H4/JCHAIN/CCL4/NOD2/CASP1/IL1R2/TLR10/CORO1A/IL29/PIK3R6/CCL1/MASP1/FCGR1A/EPX/CCN4/LEAP2/GGT5 | 81 | 13 |
| BP | OAS2/ADAR/IFI44/IFI6/ZBP1/C3/DHX58/IFIH1/NLRC5/RSAD2/THBS1/IRF7/TRIM21/IFNAR1/-/STAT2/SARM1/IGF1R/-/PARP3/-/OASL/-/IL1RL1/ZC3H12A/SLC39A10/S100A12/CXCL2/DOCK2/ENPP2/CADM1/UNC93B1/EMP2/ZBTB7B/IRF1/B2M/TRAF3IP2/PRKD2/SOCS1/TRIM13/TCIRG1/ALPK1/IRAK2/P2RX7/SMAD6/TLR4/NCKAP1L/RASGRP1/MFHAS1/BCL3/-/PLA2G6/CTSS/TEC/HFE/STING1/CARD11/CXCL11/-/S100A8/TGFB3/IL1A/-/NR1D1/TYRO3/ACOD1/-/PPARG/TNFSF15/UNC13D/SLPI/CD274/ACKR2/DDX58/CDH17/-/-/C5/HHLA2/NR1H4/JCHAIN/CCL4/NOD2/TLR10/CORO1A/IL29/CD38/PIK3R6/CD200/ANGPT1/CCL1/-/MASP1/FCGR1A/AIRE/PDCD1 | 84 | 12 |
| BP | OAS2/MOV10/IFI6/HERC5/DHX58/IFIH1/NLRC5/RSAD2/IRF7/ACP5/-/STAT2/IL22RA1/-/-/OASL/ZC3H12A/DDIT4/S100A12/LGALS8/IRF1/TRAF3IP2/P2RX7/TLR4/BCL3/PLA2G6/AGBL4/STING1/TSPAN32/UNC13D/SLPI/DDX58/-/NR1H4/JCHAIN/NOD2/IL29/FCGR1A/EPX/LEAP2 | 39 | 1 |
| CC | SOD3/LGALS3BP/C3/IGFBP2/ASAH1/-/THBS1/GREM1/HABP2/GPX3/FLRT3/SERPINB1/SORL1/HSPA8/SEMA6A/SERPINE2/SEMA4B/CCN5/CNP/KITLG/SCP2/-/CXCL2/ADA2/ENPP2/COL4A3/C1RL/MELTF/-/PROS1/COL4A4/-/GDF15/C1QTNF1/SLC12A1/CTSO/IGFBP5/-/PLA2G6/CTSS/HFE/CXCL11/-/S100A8/TGFB3/IL1A/TACSTD2/PTI/VASN/-/TNFSF15/SLC9A3/SLPI/PLTP/CD274/F12/F5/IL16/-/C5/LGI4/MATN2/CPB2/JCHAIN/EFEMP2/CCL4/IL29/ELFN2/TMPRSS6/TAFA2/AHSG/ANGPT1/SEMA6B/CCL1/ADAMTS4/-/ELFN1/MASP1/EPX/DMKN/-/CCN4 | 73 | 9 |
| CC | SOD3/LGALS3BP/C3/IGFBP2/ASAH1/-/THBS1/MICAL1/GREM1/HABP2/GPX3/FLRT3/SERPINB1/SORL1/HSPA8/SEMA6A/SERPINE2/SEMA4B/CCN5/CNP/NTN1/KITLG/-/-/SCP2/-/CXCL2/ADA2/ENPP2/COL4A3/RAB11FIP3/C1RL/CDCA8/MELTF/-/PROS1/COL4A4/FAM83D/-/GDF15/C1QTNF1/SLC12A1/CTSO/IGFBP5/-/PLA2G6/CTSS/HFE/CXCL11/-/S100A8/TGFB3/IL1A/TACSTD2/FAN1/PTI/VASN/-/NTNG2/TNFSF15/SLC9A3/SLPI/PLTP/CD274/F12/F5/IL16/-/C5/LGI4/IMPG2/MATN2/CPB2/JCHAIN/EFEMP2/CCL4/IL29/ELFN2/TMPRSS6/TAFA2/AHSG/ANGPT1/SEMA6B/CCL1/ADAMTS4/-/ELFN1/MASP1/EPX/DMKN/-/CCN4 | 79 | 13 |
| CC | LRP2/NEXN/MICAL1/FLRT3/HSPA8/SLC6A6/SARM1/IGF1R/MPST/-/MYH14/SLC9A6/FCHSD2/SPG11/DOCK4/PTPRF/STON2/SYT1/-/PDK2/IFT140/MAK/IRX3/VSIG10/DMD/LIMK1/DRD1/SHANK2/ADGRL3/NR1D1/TSPOAP1/ATP1A4/ROR1/NTNG2/KCNN2/VSTM5/NOS1/PDE6B/CPEB3/TRIM46/-/EPHA3/PCDHGB1/CORO1A/GABRG1/DNER/NPTX1/TENM2/ADCY8/KCNN3/CD200/TMIGD1/CNGA1/ARHGAP44/SYT2/ELFN1/GUCY2D/SCN2A/TULP1/USH1G/-/EPHA5/KCNC4 | 50 | 13 |
| MF | MICAL1/MSMO1/FMO5/CYP39A1/CYP7A1/-/CYP1B1/TYRP1/CYP2C42/NOS1/CYP24A1/CYP2F1/CYP8B1/CYP2W1 | 11 | 3 |
| MF | NR4A1/NKX3-1/PAQR8/HNF4G/THRA/NR1D1/PPARG/THRB/NR1H4/NR5A1 | 8 | 2 |
| MF | NR4A1/NKX3-1/THRA/NR1D1/PPARG/THRB/NR1H4/NR5A1 | 6 | 2 |
| MF | CYP39A1/CYP7A1/-/CYP1B1/CYP2C42/CYP2F1/CYP8B1 | 6 | 1 |
| MF | CYB5B/DGCR8/SUOX/CYP39A1/DUOX2/NR1D1/CYP7A1/-/CYP1B1/CYP2C42/NOS1/DUOXA2/CYP24A1/CYP2F1/CYP8B1/EPX/CYP2W1 | 13 | 4 |
| MF | C3/TAP1/-/THBS1/SYTL2/GREM1/FLRT3/-/HSPA8/DPP4/SEMA6A/SERPINE2/SEMA4B/IGF1R/CCN5/KITLG/-/LYNX1/WIPI1/NR4A1/S100A12/LGALS8/-/CXCL2/DOCK2/ADA2/COL4A3/CADM1/OSGIN1/UNC93B1/NKX3-1/EMP2/DOCK4/CHAC1/SH2B3/TRAF3IP2/FRK/SOCS1/FEM1B/GDF15/SMAD6/TLR4/SYT1/HFE/MTSS1/ASXL3/CXCL11/-/S100A8/TGFB3/IL1A/SHANK2/-/TSPOAP1/-/FGF18/PTHLH/NLGN3/PPARG/DMTN/TNFSF15/DTX1/IZUMO1R/CDH17/-/IL16/-/-/HHLA2/NR1H4/JCHAIN/CCL4/CDK5R1/IL29/DNER/TAFA2/ANGPT1/SEMA6B/CCL1/-/GMFG/-/CCN4/SRMS | 70 | 14 |
| MF | GREM1/FLRT3/DPP4/SEMA6A/SEMA4B/KITLG/-/CXCL2/OSGIN1/GDF15/CXCL11/-/TGFB3/IL1A/-/-/FGF18/PTHLH/TNFSF15/IL16/-/CCL4/IL29/TAFA2/SEMA6B/CCL1/-/GMFG | 22 | 6 |
| MF | NR4A1/NKX3-1/THRA/PPARG/THRB/NR1H4/NR5A1 | 6 | 1 |
| MF | SCD/MICAL1/MSMO1/SC5D/FMO5/RIOX1/CYP39A1/-/CYP7A1/-/CYP1B1/TYRP1/CYP2C42/NOS1/CYP24A1/CYP2F1/CYP8B1/CYP2W1 | 14 | 4 |
| MF | GREM1/FLRT3/DPP4/SEMA6A/SEMA4B/KITLG/LYNX1/-/CXCL2/OSGIN1/GDF15/CXCL11/-/TGFB3/IL1A/-/-/FGF18/PTHLH/TNFSF15/IL16/-/CCL4/IL29/TAFA2/SEMA6B/CCL1/-/GMFG | 23 | 6 |
| MF | CELSR1/LRP2/THBS1/HABP2/GNPTAB/EHD3/CDH6/PLCB1/-/S100A12/ENPP2/RAB11FIP3/SUSD1/PROS1/PCDHGA4/GCA/SYT1/RASGRP1/TBC1D8B/THBD/DUOX2/S100A8/ADGRL3/CRB2/DSG3/RASGRP3/CDHR5/F12/CDH17/DUOXA2/PAMR1/-/PCDHGB1/MATN2/EFEMP2/CAPSL/PLCB2/CAPS2/DNAH7/DNER/TENM2/LPCAT2/TNNT2/CDH4/SYT2/GUCA1B/TNNT1/MASP1/PLCH1/DOC2B | 44 | 6 |
| MF | GREM1/KITLG/CXCL2/GDF15/CXCL11/-/TGFB3/IL1A/-/-/TNFSF15/IL16/-/CCL4/IL29/CCL1 | 13 | 3 |
| MF | CYB5B/DGCR8/SUOX/CYP39A1/DUOX2/NR1D1/CYP7A1/-/CYP1B1/CYP2C42/NOS1/DUOXA2/CYP24A1/CYP2F1/CYP8B1/EPX/CYP2W1 | 13 | 4 |

**Supplementary Table** 16 GO enrichment results of S36 vs Control.

| Category | Gene name | Up-regulated number | Down-regulated number |
| --- | --- | --- | --- |
| BP | TFRC/OAS2/IFI44/IFI6/ZBP1/DHX58/C3/IRF7/-/OASL/RSAD2/ADAR/-/B2M/NLRC5/-/TRIM21/CXCL2/IL1RL1/UNC93B1/ENPP2/DOCK2/ZC3H12A/-/IL1A/SLC39A10/IFIH1/STAT2/IRF1/UNG/RAB29/CTSS/S100A12/MYB/ZBTB7B/IFNAR1/MFHAS1/-/HFE/PRKD2/SARM1/LCP2/CXCL11/TEC/SOCS1/-/CXCL8/TCIRG1/TYRO3/BCL3/RASGRP1/S100A8/ALPK1/TNIP3/ACOD1/CARD11/SMAD6/TLR10/-/ENPP3/STING1/NR1D1/CDH17/ACKR2/C5/-/HHLA2/TNFSF15/CD38/-/CD274/LAX1/-/P2RX7/TGFB3/SLPI/CCL1/IL5/ITK/POU2F2/CD200/IL29/NOD2/ANGPT1/FCRLB/PIK3R6/NOS2/PRNP/PDCD1/CORO1A/MASP1/SEMA4A/SLC46A2/PGLYRP2/- | 85 | 10 |
| BP | OAS2/IFI6/ZBP1/HERC5/DHX58/C3/IRF7/-/OASL/TAP1/DDIT4/MOV10/RSAD2/ADAR/-/NLRC5/-/LDLR/TRIM21/CEBPB/DPP4/CXCL2/IL1RL1/UNC93B1/ACP5/ZC3H12A/IL1A/ZFP36/IFIH1/STAT2/IRF1/IL22RA1/S100A12/SYT11/ELF3/IFNAR1/MFHAS1/RNF26/SOCS3/TRAF3IP1/DUOX2/-/SARM1/CXCL11/SOCS1/MAPKBP1/CXCL8/TCIRG1/RB1/TYRO3/BCL3/RASGRP1/PTGFR/S100A8/ALPK1/TNIP3/NCBP3/ACOD1/CPSF6/TLR10/ENPP3/STING1/DUOXA2/NR1D1/ACKR2/NR2E1/C5/CASP1/-/CCN3/-/F12/-/-/P2RX7/SLPI/CCL1/IL1R2/IL29/NOD2/PIK3R6/NOS2/CORO1A/MASP1/EPX/SLC46A2/PGLYRP2 | 75 | 12 |
| BP | DHX58/IRF7/RSAD2/UNC93B1/IFIH1/IRF1/MFHAS1/SARM1/TYRO3/ALPK1/TNIP3/TLR10/STING1/NR1D1/-/NOD2/SLC46A2 | 13 | 4 |
| CC | TFRC/LGALS3BP/C3/CNP/-/-/VEGFA/IGFBP2/HSPA8/SOD3/ASAH1/HABP2/FLRT3/CXCL2/SERPINB1/GDF15/MELTF/ENPP2/NTN1/SETX/IL1A/MICAL1/ADA2/SEMA4B/KITLG/PTPRZ1/MTCL1/GPX3/SLC11A2/AREG/-/RAB29/TACSTD2/CTSS/FAM83D/NTN4/FAN1/CCN5/C1RL/-/WNT7B/RTN4RL1/SCP2/SOGA1/HFE/CXCL11/-/CXCL8/SAAL1/CTSO/S100A8/SLC12A1/C1QTNF1/PPP1R13L/IGFBP5/IL16/TIMP4/PTI/C5/CCN3/SLC9A3/PLTP/NTNG2/TNFSF15/-/-/CD274/MGP/F12/EFEMP2/IMPG2/TGFB3/SLPI/CCL1/IL5/MMP24/SEMA6B/CCN6/CPB2/IL29/ANGPT1/LGI4/TAFA2/DMKN/ADAMTS4/-/OLFML2B/MASP1/EPX/SNRNP25/FBLN1/SEMA4A/- | 74 | 19 |
| CC | TFRC/LGALS3BP/C3/CNP/VEGFA/IGFBP2/HSPA8/SOD3/ASAH1/HABP2/FLRT3/CXCL2/SERPINB1/GDF15/MELTF/ENPP2/IL1A/ADA2/SEMA4B/KITLG/MTCL1/GPX3/AREG/-/TACSTD2/CTSS/CCN5/C1RL/-/WNT7B/RTN4RL1/SCP2/SOGA1/HFE/CXCL11/-/CXCL8/SAAL1/CTSO/S100A8/SLC12A1/C1QTNF1/IGFBP5/IL16/TIMP4/PTI/C5/SLC9A3/PLTP/TNFSF15/-/-/CD274/F12/EFEMP2/TGFB3/SLPI/CCL1/IL5/MMP24/SEMA6B/CCN6/CPB2/IL29/ANGPT1/LGI4/TAFA2/DMKN/ADAMTS4/-/MASP1/EPX/FBLN1/SEMA4A/- | 63 | 12 |

**Supplementary Table** 17 KEGG pathway enrichment results of DEGs in S6 vs Control.

| KEGG ID | Description | Gene Name |
| --- | --- | --- |
| ssc05164 | Influenza A | MX1/OAS2/IRF7/IFIH1/OAS1/SOCS3/RSAD2 |
| ssc05160 | Hepatitis C | MX1/OAS2/IFIT1/IRF7/OAS1/SOCS3/RSAD2 |
| ssc04622 | RIG-I-like receptor signaling pathway | ISG15/DHX58/IRF7/IFIH1 |
| ssc05162 | Measles | MX1/OAS2/IRF7/IFIH1/OAS1 |
| ssc04668 | TNF signaling pathway | CXCL2/IL1RL1/SOCS3 |
| ssc04061 | Viral protein interaction with cytokine and cytokine receptor | CXCL2/IL1RL1 |
| ssc04621 | NOD-like receptor signaling pathway | OAS2/CXCL2/IRF7/OAS1 |

**Supplementary Table** 18 KEGG pathway enrichment results of DEGs in S12 vs Control.

| KEGG ID | Description | Gene Name |
| --- | --- | --- |
| ssc05164 | Influenza A | OAS2/MX1/ADAR/STAT1/PML/TRIM25/RSAD2/IFIH1/OAS1/GHSR/NLRP3/TLR4/SOCS3/CXCL10/DDX58/TMPRSS2 |
| ssc04668 | TNF signaling pathway | CXCL2/IL1RL1/CREB5/PTGS2/SOCS3/CXCL10/MAP2K6/TNFAIP3/CSF2/VCAM1 |
| ssc05160 | Hepatitis C | OAS2/MYC/MX1/STAT1/IFIT1/RSAD2/CLDN8/OAS1/SOCS3/CLDN6/CXCL10/CLDN2/DDX58/CLDN9 |
| ssc00140 | Steroid hormone biosynthesis | CYP1A1/CYP1B1/AKR1C2/HSD17B6/CYP1A2 |
| ssc04657 | IL-17 signaling pathway | FOSL1/CXCL2/MMP7/PTGS2/CXCL10/TNFAIP3/S100A8/CSF2 |
| ssc00830 | Retinol metabolism | CYP1A1/CYP26A1/HSD17B6/CYP1A2 |
| ssc05204 | Chemical carcinogenesis | CYP1A1/PTGS2/SULT2A1/CYP1B1/CYP1A2 |
| ssc05162 | Measles | OAS2/MX1/ADAR/STAT1/HSPA8/IFIH1/OAS1/TLR4/TNFAIP3/DDX58 |
| ssc04061 | Viral protein interaction with cytokine and cytokine receptor | GHSR/CXCL2/IL1RL1/IL22RA1/CXCL10/CCL4/CCL1 |
| ssc04621 | NOD-like receptor signaling pathway | OAS2/STAT1/OAS1/CXCL2/NLRP3/TLR4/MNDA/TNFAIP3 |
| ssc05323 | Rheumatoid arthritis | CXCL2/MMP7/TLR4/TCIRG1/CSF2/CCL4 |

**Supplementary Table** 19 KEGG pathway enrichment results of DEGs in S18 vs Control.

| KEGG ID | Description | Gene Name |
| --- | --- | --- |
| ssc05323 | Rheumatoid arthritis | MMP7/ACP5/CXCL2/ATP6V1B1/CD86/FOS/TLR4/IL15/TCIRG1/IL1A/HLA-DOB/CSF2/CD80/SLA-DMA/TNFSF13B/TNFRSF11A |
| ssc05164 | Influenza A | STAT1/MX1/OAS2/ADAR/PML/RSAD2/TRIM25/OAS1/GHSR/IRF7/IFIH1/RNASEL/CASP7/STAT2/NLRP3/CXCL10/TLR4/KPNA5/SOCS3/TMPRSS2/IL1A/HLA-DOB/IRF9/SLA-DMA/IFNB1 |

**Supplementary Table** 20 KEGG pathway enrichment results of DEGs in S24 vs Control.

| KEGG ID | Description | Gene Name |
| --- | --- | --- |
| ssc05323 | Rheumatoid arthritis | MMP7/ACP5/CD86/FOS/ATP6V1B1/CXCL2/TCIRG1/TNFSF13B/TLR4/IL15/TGFB3/IL1A/ITGAL/HLA-DOB/CCL5/ATP6V1C2/CCL4/CD80/ANGPT1/SLA-DMA/SLA-DMB/CSF2 |
| ssc05164 | Influenza A | STAT1/MX1/OAS2/ADAR/TRIM25/PML/GHSR/RNASEL/IFIH1/OAS1/RSAD2/IRF7/STAT2/CASP7/NFKBIA/CXCL10/NLRP3/FDPS/TLR4/SOCS3/KPNA5/IL1A/TMPRSS2/IRF9/HLA-DOB/DDX58/CCL5/CASP1/KLK11/IFNB1/SLA-DMA/SLA-DMB/IFN-ALPHAOMEGA |
| ssc05332 | Graft-versus-host disease | CD86/IL1A/HLA-DOB/CD80/SLA-DMA/SLA-DMB |
| ssc05150 | Staphylococcus aureus infection | C3/ITGAL/HLA-DOB/C5/C2/MASP2/SLA-DMA/SLA-DMB/MASP1/FCGR1A/KRT23 |
| ssc04514 | Cell adhesion molecules (CAMs) | CD86/CLDN8/SDC3/CADM1/PTPRF/CLDN6/SIGLEC1/CLDN2/ITGAL/ALCAM/NLGN3/HLA-DOB/NTNG2/NCAM2/CD274/CD80/SLA-DMA/TIGIT/NRCAM/CDH4/SLA-DMB/CLDN16/PDCD1 |
| ssc04610 | Complement and coagulation cascades | C3/SERPING1/PROS1/THBD/PROC/F12/F5/C5/F10/C2/CPB2/MASP2/F2/C4BPB/MASP1 |
| ssc04621 | NOD-like receptor signaling pathway | STAT1/OAS2/RNASEL/OAS1/IRF7/STAT2/NAMPT/MNDA/GBP2/PLCB1/NFKBIA/CXCL2/RIPK2/NLRP3/P2RX7/TLR4/ANTXR1/STING1/IRF9/CYBA/CCL5/NOD2/CASP1/PLCB2/IFNB1/IFN-ALPHAOMEGA/TRPM2 |
| ssc04978 | Mineral absorption | SLC34A1/SLC31A1/TRPV5/MT1A/SLC5A1/ATP2B2/SLC30A1/ATP1A4/TRPM6/SLC9A3/TF/ATP2B3/SLC8A2 |
| ssc04940 | Type I diabetes mellitus | CD86/IL1A/HLA-DOB/CD80/SLA-DMA/PTPRN/SLA-DMB |
| ssc05320 | Autoimmune thyroid disease | CD86/HLA-DOB/CD80/SLA-DMA/SLA-DMB/IFN-ALPHAOMEGA |
| ssc05133 | Pertussis | C3/SERPING1/CFL2/CASP7/FOS/CALML4/IRF1/GNAI1/NLRP3/TLR4/IL1A/LY96/C5/C2/CASP1/C4BPB |
| ssc05330 | Allograft rejection | CD86/HLA-DOB/CD80/SLA-DMA/SLA-DMB |
| ssc04672 | Intestinal immune network for IgA production | PIGR/CD86/TNFSF13B/IL15/HLA-DOB/CD80/SLA-DMA/SLA-DMB |
| ssc03320 | PPAR signaling pathway | SCD/PCK1/HMGCS1/FADS2/MMP7/SCP2/PLIN2/OLR1/FABP3/CYP7A1/PPARG/PLTP/SLC27A2/PLIN5/CYP8B1/SCD5 |
| ssc04612 | Antigen processing and presentation | ZBTB22/HSPA8/RFX5/CTSS/HLA-DOB/PSME1/SLA-DMA/SLA-DMB |

**Supplementary Table** 21 KEGG pathway enrichment results of DEGs in S36 vs Control.

| KEGG ID | Description | Gene Name |
| --- | --- | --- |
| ssc05323 | Rheumatoid arthritis | FOS/VEGFA/MMP7/CXCL2/CD86/ACP5/IL1A/TNFSF13B/ATP6V1B1/ATP6V1C2/TCIRG1/ITGAL/IL15/CCL5/IL18/HLA-DOB/CD80/TGFB3/ATP6V0A4/ANGPT1/HLA-DRA |
| ssc00100 | Steroid biosynthesis | MSMO1/FDFT1/SC5D/SQLE/CYP51A1/DHCR24/  LIPA/DHCR7/TM7SF2/CYP24A1 |
| ssc04514 | Cell adhesion molecules (CAMs) | ITGAV/CD86/SIGLEC1/SDC3/CLDN8/ITGAL/ALCAM/IGSF11/NCAM2/NTNG2/HLA-DOB/CD274/CD80/NLGN3/NRCAM/CLDN2/CNTNAP1/ESAM/CD4/PDCD1/NRXN3/CLDN16/HLA-DRA |
| ssc04621 | NOD-like receptor signaling pathway | STAT1/OAS2/IRF7/OAS1/NAMPT/NFKBIA/CXCL2/RNASEL/PLCB1/STAT2/GBP2/MNDA/ANTXR1/RIPK2/MAPK11/CCL5/STING1/CASP1/IL18/IRF9/PLCB2/P2RX7/NOD2/TRPV2 |
| ssc05133 | Pertussis | C3/SERPING1/FOS/CALM1/IL1A/IRF1/GNAI1/CASP7/CALML4/MAPK11/C2/C5/CASP1/TMEM52/C4BPB/NOS2 |
| ssc05150 | Staphylococcus aureus infection | C3/ITGAL/C2/C5/HLA-DOB/KRT10/MASP1/HLA-DRA |
| ssc04672 | Intestinal immune network for IgA production | PIGR/CD86/TNFSF13B/IL15/HLA-DOB/CD80/IL5/HLA-DRA |


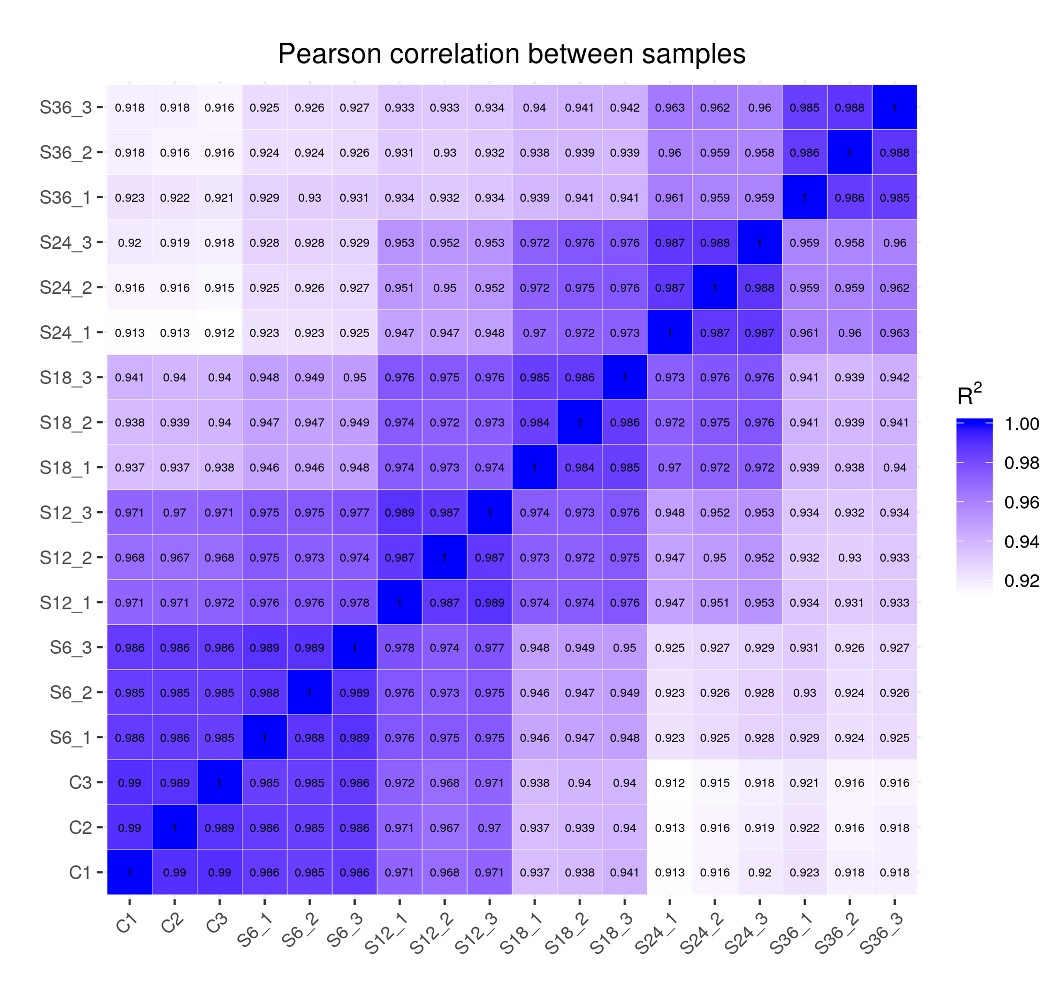


**Supplementary Fig.1 Analysis of sample correlation.** Heatmap shows the correlation coefficient (R^2^) of gene expression levels between samples. The dark purple of the heatmap represented the high correlation and the light purple represented the low correlation. C1-3: control groups; S6_1-3: PK-15 cells at 6 h SVA post infection groups; S12_1-3: PK-15 cells at 12 h SVA post infection groups; S18_1-3: PK-15 cells at 18 h SVA post infection groups; S24_1-3: PK-15 cells at 24 h SVA post infection groups; S36_1-3: PK-15 cells at 36 h SVA post infection groups.
