# Supplementary figures and images for: Transcriptome Analyses of Senecavirus A-Infected PK-15 Cells: RIG-I and IRF7 Are the Important Factors in Inducing Type III Interferons
Source: Front Microbiol. 2022 Mar 4;13:846343. doi: 10.3389/fmicb.2022.846343 (PMC8931416; doi:10.3389/fmicb.2022.846343)

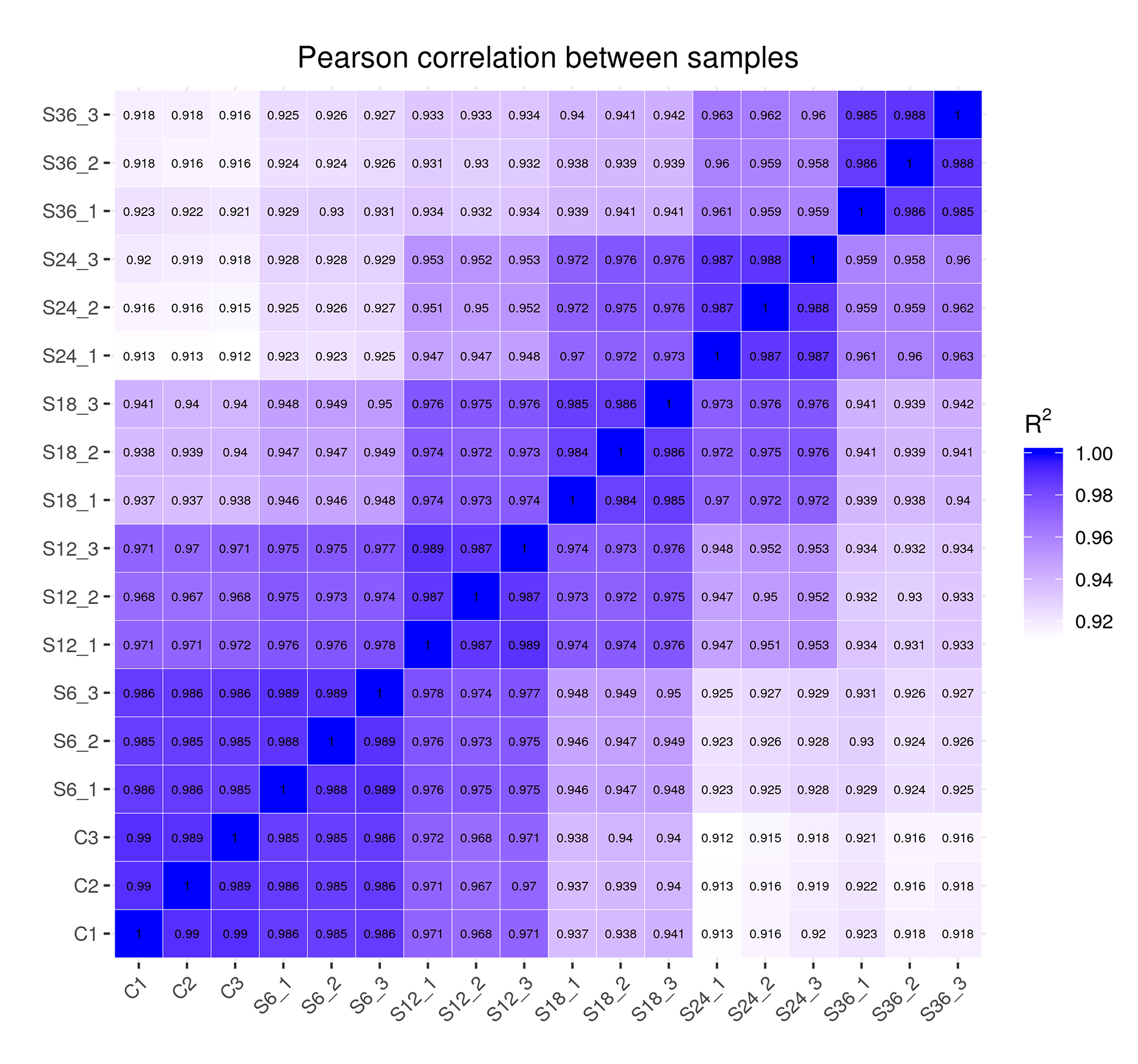

Supplement: Supplementary file 1 [file Data_Sheet_1.zip › Supplementary data/Supplementary figure 1.tif]
